# Chelate-conjugated β-lactams resensitize metallo-β-lactamase IMP-1-producing gram-negative bacteria against carbapenem antibiotics

**DOI:** 10.1128/spectrum.02532-25

**Published:** 2026-07-23

**Authors:** Touya Toyomoto, Katsuhiko Ono, Hina Honda, Tianli Zhang, Hiroyasu Tsutsuki, Ayaka Uegama, Rika Fukushima, Keiichi Yamamoto, Yasuhito Tanaka, Kanyawee Nakburee, Worasak Kaewkong, Hitoshi Tsugawa, Tomohiro Sawa

**Affiliations:** 1 Department of Microbiology, Graduate School of Medical Sciences, Kumamoto University 13205, Kumamoto, Japan; 2 Transkingdom Signaling Research Unit, Division of Host Defense Mechanism, Tokai University School of Medicine 38261, Isehara, Japan; 3 Comprehensive Center for Infectious Disease Control, Akita University Graduate School of Medicine 12934 , Akita, Japan; 4 Department of Clinical Microbiology, Faculty of Life Sciences, Kumamoto University 13205, Kumamoto, Japan; 5 Department of Gastroenterology and Hepatology, Graduate School of Medical Sciences, Kumamoto University 13205, Kumamoto, Japan; 6 Department of Microbiology, Faculty of Medical Science, Naresuan University 59212, Phitsanulok, Thailand; 7 Department of Biochemistry, Faculty of Medical Science, Naresuan University, Phitsanulok, Thailand; Illinois State University, Normal, Illinois, USA

**Keywords:** metallo-β-lactamase, antimicrobial resistance, carbapenem, chelator hybrid, MBL inhibitor, IMP-1, VIM-2

## Abstract

Metallo-β-lactamases (MBLs) such as IMP-1 are zinc-dependent enzymes that degrade a wide range of β-lactam antibiotics, including carbapenems. Although many efforts have been made to develop MBL inhibitors, there are no approved inhibitors to date. Polycarboxylated amoxicillin, such as diethylenetriaminepentaacetic acid (DTPA)-conjugated amoxicillin, has been developed as a potent antipseudomonal antibiotic. Interestingly, it effectively killed *Pseudomonas aeruginosa* clinical isolates that expressed IMP-1. We hypothesized that DTPA-amoxicillin gained activity as an MBL inhibitor due to its metal-chelating ability. Here, we investigated the inhibitory activities of DTPA-conjugated β-lactams (DTPA-β-lactams) against MBL. We synthesized a series of DTPA-β-lactams and evaluated their inhibitory activity against IMP-1 and VIM-2. Almost all DTPA-β-lactams exhibited potent inhibitory activity against IMP-1 and VIM-2. Notably, these DTPA-β-lactams remarkably resensitized IMP-1-expressing bacteria, including *Klebsiella pneumoniae*, *Escherichia coli*, *Citrobacter amalonaticus*, and *C. freundii*, against carbapenem. In *in vivo* mouse models, a representative DTPA-β-lactam pretreatment drastically enhanced the therapeutic effect of meropenem against lethal infections by IMP-1-expressing bacteria. These observations warrant further investigation of chelator-conjugated β-lactam as new MBL inhibitors to treat carbapenem-resistant bacterial infections.

**IMPORTANCE:**

The global rise of antibiotic-resistant bacteria has become a serious threat to public health. Among them, gram-negative bacteria harboring metallo-β-lactamase (MBL) genes, which inactivate carbapenems—the last-resort antibiotics—pose a particular challenge due to the lack of effective therapeutic options. In this study, we developed β-lactam-based compounds incorporating a metal-chelating moiety designed to inhibit MBL. These compounds demonstrated potent inhibitory activity against MBL. Moreover, when combined with carbapenems, they significantly restored antibacterial efficacy in MBL-producing strains. Despite their structural simplicity, the compounds offer substantial flexibility in chemical modification, allowing for tailored optimization against different resistant pathogens. These findings suggest that such inhibitors could serve as promising candidates for novel combination therapies targeting MBL-mediated resistance, contributing to the development of next-generation antimicrobial strategies.

## INTRODUCTION

Urgent action is needed to address the growing threat of antimicrobial resistance (AMR). Recent estimates suggest that 1.27 million deaths are directly attributable to bacterial antibiotic resistance (1). If no action is taken, AMR is projected to cause 10 million deaths annually and become the leading cause of mortality, surpassing cancer, by 2050 (2).

Carbapenems are a class of β-lactam antibiotics that inhibit bacterial growth by disrupting cell wall synthesis (3). β-lactam antibiotics are the most widely used class of antibiotics, accounting for 65% of injectable antibiotics used in the United States (4). However, these lifesaving drugs are sometimes ineffective against bacterial infections because bacteria employ several resistance mechanisms, including low permeability, efflux pump expression, mutations in penicillin-binding proteins, and the production of β-lactamases (5).

Carbapenem resistance in Enterobacterales and other gram-negative bacteria is primarily mediated by the production of carbapenemases, which include both serine-carbapenemases (SBLs) and metallo-β-lactamases (MBLs). Globally, KPC-type SBLs are among the most prevalent carbapenemases in many regions, whereas MBLs are increasingly disseminating and represent a major clinical concern (6–10). Imipenemase-1 (IMP-1) is an MBL that degrades β-lactam antibiotics and hence confers almost all of β-lactam resistance except for the monobactam group (11). In clinical settings, combination therapies such as ceftazidime/avibactam and aztreonam/cefiderocol are recommended for the treatment of MBL-expressing Gram-negative bacteria, including Enterobacterales, *Pseudomonas aeruginosa*, and *Stenotrophomonas maltophilia* (12–14). However, accumulating evidence suggests that treatment failure rates for infections caused by MBL-producing pathogens remain high because of their broad-spectrum antibiotic resistance, thereby limiting available therapeutic options. This highlights the urgent need for the development of effective MBL inhibitors. To date, extensive efforts have been made to develop MBL inhibitors; however, no clinically approved MBL inhibitors are currently available (15). This is likely due to the challenges associated with inhibitor design, particularly the intrinsic properties of zinc coordination chemistry and the structural diversity of MBL active sites (16).

MBL inhibitors reported to date can be broadly categorized into five groups, including thiol-based inhibitors, carboxylate-containing molecules, cyclic boronates, β-lactam analogs, and metal chelators (17). The first four classes typically interact with the active-site zinc ions through thiol, carboxylate, or boronate functionalities or by mimicking β-lactam substrates. While these inhibitors have provided valuable mechanistic insights, only a limited number have advanced to clinical use. In contrast, Type V inhibitors, represented by metal chelators such as ethylenediaminetetraacetic acid (EDTA) and diethylenetriaminepentaacetic acid (DTPA), function by sequestering zinc and thereby inactivating MBL (18). Although conventional chelators suffer from a lack of selectivity and systemic toxicity, recent efforts have explored chemical modifications and conjugation strategies to enhance their local activity and reduce off-target effects. We have previously developed DTPA-conjugated amoxicillin (DTPA-AMOX) as a potent antipseudomonal drug (19). Interestingly, the drug was effective against clinically isolated *P. aeruginosa* strains which expressed IMP-1 and resistance to all β-lactam antibiotics, including carbapenems (19). As mentioned earlier, metal chelators can act as Type V MBL inhibitors. We therefore hypothesize that the DTPA moiety of the drug may function as a metal chelator to inhibit MBL. In order to prove this hypothesis, we investigated in this study the inhibitory activities of DTPA-conjugated β-lactams (DTPA-β-lactams) against MBL. Our data suggested that DTPA-β-lactams act as potent inhibitors against MBLs including IMP-1 and verona integron-encoded metallo-β-lactamase 2 (VIM-2). More importantly, DTPA-β-lactams successfully resensitize IMP-1-expressing Enterobacterales such as *Escherichia coli* and *Klebsiella pneumoniae* against carbapenems. We further demonstrated that a representative DTPA-β-lactam significantly enhanced the therapeutic action of carbapenem *in vivo* against lethal infection of IMP-1-expressing *K. pneumoniae*. These data indicated that the combination of carbapenems with chelator-conjugated β-lactams may become a new option for the treatment of MBL-expressing bacterial infections.

## MATERIALS AND METHODS

### Reagents

AMOX trihydrate, ampicillin (AMP) sodium, meropenem (MEPM) trihydrate, imipenem (IPM) trihydrate, cefalexin (CFLX), isopropyl-β-d-(−)-thiogalactopyranoside (IPTG), and formic acid were purchased from Fujifilm Wako Pure Chemical Co., Ltd. (Osaka, Japan). Cefadroxil (CFDX) was purchased from MP Biomedicals (Irvine, CA, USA). DTPA dianhydride, ethylenediaminetetraacetic acid (EDTA) dianhydride, 7-aminodesacetoxycephalosporanic acid (ADCA), and cefaclor (CFCL) monohydrate were purchased from Tokyo Chemical Industry Co., Ltd. (Tokyo, Japan). Taniborbactam (TNBM) hydrochloride was purchased from MedChemExpress (Monmouth Junction, NJ, USA). Sodium chloride (NaCl) was purchased from Nacalai Tesque (Kyoto, Japan), peptone was purchased from Nihon Pharmaceutical Co., Ltd. (Osaka, Japan), yeast extract was purchased from Oriental Yeast Company, Ltd. (Tokyo, Japan), and acetonitrile was purchased from Kanto Chemical Co., Inc. (Tokyo, Japan), respectively.

### Bacterial strains

Clinical isolates, including *K. pneumoniae* strains KP1 and KP2, *Citrobacter amalonaticus*, *C. freundii*, *E. coli*, and VIM-2-expressing *P. aeruginosa*, were provided by Kumamoto University Hospital. *E. coli* strains DH 5α and BL21(DE3) pLysS were obtained from laboratory stock, and *bla*_IMP-1_- or *bla*_VIM-2_-transformed DH 5α and BL21(DE3) pLysS strains were constructed in this study. All bacterial strains used in this study are summarized in Table S1.

### Generation of His-tagged IMP-1 and VIM-2

Plasmids encoding *bla*_IMP-1_ and *bla*_VIM-2_ were isolated from KP1 and clinically isolated *P. aeruginosa*, respectively. The *bla*_IMP-1_ and *bla*_VIM-2_ were amplified by polymerase chain reaction (PCR) using KOD-plus-Neo (Toyobo, Osaka, Japan) with the isolated plasmids as a template and the following primers: *Bam*HI-IMP-1-forward (5′-GGGGGATCCATGGCAGAGTCTTTGCCAGA-3′) and *Pst*I-IMP-1-reverse (5′-CCCCTGCAGGTTAGAAAATTTAGTTGC-3′) for *bla*_IMP-1_; and, *Bam*HI-VIM-2-forward (5′-GGGGGATCCATGAGTCCGCTCGCTTTTTC-3′) and *Pst*I-VIM-2-reverse (5′-CCCCTGCAGCTACTCAACGACTGAGCGATT-3′) for *bla*_VIM-2_. The amplified DNA fragments were inserted in-frame downstream of the N-terminal 6 × His tag in pQE80L (Qiagen, Hilden, Germany), generating pQE-IMP-1 or pQE-VIM-2 encoding N-terminal His-tagged IMP-1 and VIM-2, respectively. *E. coli* BL21(DE3) pLysS was transformed with pQE-IMP-1 or pQE-VIM-2 for protein expression. Overnight cultures of *E. coli* BL21(DE3) pLysS harboring pQE-IMP-1 or pQE-VIM-2 were diluted 100-fold in LB medium supplemented with 100 μg/mL AMP and then cultured at 37°C with shaking until the optical density at 600 nm reached 0.5–0.6. After 3 h of induction with 0.2 mM IPTG, the cultures were centrifuged to collect bacterial pellets. The pellets were washed with PBS and then suspended in lysis buffer (50 mM NaH_2_PO_4_ [pH 8.0], 300 mM NaCl, and 10 mM imidazole). The bacterial suspensions were sonicated, and then the supernatants were collected. The supernatants were applied to Ni-NTA agarose column (Qiagen), and the column was washed three times with wash buffer (50 mM NaH_2_PO_4_ [pH 8.0], 300 mM NaCl, 20 mM imidazole, and 10% glycerol). His-tagged IMP-1 or His-tagged VIM-2 was eluted by elution buffer (buffer 1: 50 mM NaH_2_PO_4_ [pH 8.0], 300 mM NaCl, 100 mM imidazole, and 10% glycerol; buffer 2: 50 mM NaH_2_PO_4_ [pH 8.0], 300 mM NaCl, 500 mM imidazole, and 10% glycerol) and the sample was then subjected to gel filtration using PD-10 desalting column (Cytiva, MA, USA) to replace the solvent with storage buffer (50 mM Tris-HCl pH 7.5 supplemented with 0.5 M NaCl). Purified proteins were analyzed by sodium dodecyl sulfate (SDS)-polyacrylamide gel electrophoresis (PAGE). All plasmids used or constructed in this study are summarized in Table S1.

### PCR determination of IMP-1

DNA of clinical isolates was extracted by using Cica Geneus DNA Extraction Reagent (Kanto Chemical Co., Inc). PCR determination of *bla*_IMP-1_ was performed by using KOD FX (Toyobo) with the primers described above (BamHI-IMP-1-F and PstI-IMP-1-R), according to the manufacturer’s instructions.

### Syntheses of DTPA-β-lactams

DTPA-β-lactams were synthesized by reacting β-lactam antibiotics with DTPA anhydrides in 0.4 M Na_2_HPO_4_ at 37°C for 30 min. Reaction mixtures were subjected to open column chromatography with Wakogel 100C18 (Fujifilm Wako Pure Chemical Co., Ltd.). Mobile phases A (0.1% formic acid) and B (acetonitrile) were used with the following gradient: for DTPA-ADCA, B was set at 0%, 0.5%, 8%, 12%, 14%, and 50%; for DTPA-AMOX, B was set at 0%, 4%, 8%, 12%, 20%, 25%, 30%, and 35%. Each fraction was monitored by means of LC-electrospray ionization (ESI)-MS to confirm the elution of DTPA-β-lactam. The fractions of DTPA-AMP, DTPA-CFLX, DTPA-CFCL, and DTPA-CFDX were collected by means of HPLC under the conditions mentioned below. The fractions containing the DTPA-β-lactam were lyophilized and then further purified by HPLC as described below.

### HPLC

Lyophilized fractions containing DTPA-β-lactams were subjected to reverse-phase HPLC purification with YMC-Pack ODS-AQ (4.6 × 250 mm; YMC Co., Ltd., Kyoto, Japan). HPLC separation was performed by using the Agilent 1260 Infinity series equipped with a photodiode array detector and an automated fraction collector (Agilent Technologies, Santa Clara, CA, USA). Samples were separated at 35°C. Mobile phases A (0.1% formic acid) and B (acetonitrile) were used with a linear gradient from 0.5% to 40% B for 15 min, with the gradient maintained at 40% B for 1 min, after which the gradient was decreased to 0.5% B in 1 min, with a flow rate of 0.8 mL/min. All compounds were detected at 254 nm. Peaks containing DTPA-β-lactams were collected.

### LC-MS/MS

Determination of DTPA-β-lactam formation by measuring the corresponding molecular weights and IMP-1 inhibitory assays based on MEPM degradation was performed by using LC-ESI-MS with the Agilent 6460 Triple Quadrupole LC/MS system (Agilent Technologies). The analytical conditions were as follows: column, YMC-Triart C18 Plus column (2.1 × 50 mm) (YMC Co., Ltd.); column temperature, 45°C; injection volume, 1 μL; mobile phases: A, 0.1% formic acid, and B, acetonitrile; gradient (B concentration), 0 min–0.5%, 10 min–80%, 10.1 min–0.5%, 15 min–0.5%; and flow rate, 0.2 mL/min. General conditions used for electrospray ionization-MS were as follows: nebulizer gas, nitrogen delivered at 50 psi; nebulizer gas temperature, 250°C; capillary voltage, 3,500 V; collision gas, G1 grade nitrogen (Taiyo Nippon Sanso Corp. Tokyo, Japan).

### LC-MS/MS determination of MEPM degradation for IMP-1 inhibition assay

The enzymatic activity of His-tagged IMP-1 and VIM-2 and their inhibition by DTPA-β-lactams were determined by monitoring the degradation of MEPM by means of LC-MS/MS. In detail, 100 μM DTPA-ADCA and various concentrations of zinc sulfate were pre-incubated in a reaction buffer containing 50 mM HEPES-NaOH (pH 7.5) and 200 mM NaCl at 30°C for 10 min. The recombinant IMP-1 (300 nM) or VIM-2 (300 nM) was added to a reaction buffer, and then incubation at 30°C for 10 min. The mixture was added to a final concentration of 100 µM MEPM, and the reaction was continued for an additional 30 min at 30°C. The samples were diluted 100-fold with water and then subjected to LC-MS/MS analysis using multiple reaction monitoring mode. The parameters for MEPM detection were as follows: MEPM precursor ion (*m/z*), 384.2; product ion (*m/z*), 68; fragmentor voltage (V), 90; collision energy, 49; and polarity, positive.

### Spectrophotometric determination of IPM degradation for IMP-1 inhibition assay

The enzymatic activity of IMP-1 and VIM-2 was measured with slight modification to the method described by Wachino et al. (20). Briefly, 100 nM His-tagged IMP-1 or VIM-2 was pre-incubated with various concentrations of test reagents in 50 mM HEPES-NaOH buffer (pH 7.5) supplemented with 200 mM NaCl at 30°C for 5 min, followed by the addition of 200 μM IPM. Enzyme activity was determined by monitoring the changes in absorbance at 298 nm for 1 min, which reflects IPM degradation.

### Zinc quantitation assay

The zinc contents of IMP-1 and VIM-2, with or without DTPA-ADCA treatment, were determined using a Metalloassay kit (Metallogenics Co., Ltd., Chiba, Japan) according to the manufacturer’s instructions. Briefly, 10 µM IMP-1 or VIM-2 was incubated with 100 µM DTPA-ADCA in 100 mM HEPES buffer (pH 7.5) for 10 min at room temperature. In this experiment, NaCl-free buffer was used in order to avoid interference of the salt with the zinc measurement. Free DTPA-ADCA and zinc were removed using a PD MiniTrap G-25 column (Cytiva, Tokyo, Japan). The flow-through fractions containing IMP-1 or VIM-2 were then subjected to the Metalloassay kit to determine zinc concentration.

### Determination of inhibition constants for DTPA-ADCA against IMP-1 and VIM-2

The inhibition constants (*K*_i_) of DTPA-ADCA against IMP-1 and VIM-2 were determined using Dixon plots. IMP-1 or VIM-2 (100 nM) was incubated with DTPA-ADCA at concentrations ranging from 0 to 50 µM for IMP-1 and from 0 to 0.1 µM for VIM-2 in 50 mM HEPES buffer (pH 7.5) containing 200 mM NaCl. After incubation at 30°C for 10 min, enzyme activity was measured using the spectrophotometric assay described above with IPM concentrations of 10, 20, and 40 µM. Data were obtained from at least two independent experiments.

### Determination of minimum inhibitory concentration (MIC) of MEPM against clinical isolates expressing IMP-1

Clinical isolates that express IMP-1 were cultured overnight and then diluted 1,000-fold in LB medium. The suspensions thus obtained were plated in a 96-well plate and were treated with various concentrations of MEPM. Bacterial growth was measured by 655 nm absorbance after 18–20 h of incubation at 37°C.

### Effects of DTPA-β-lactams on meropenem susceptibility of clinical isolates expressing IMP-1

Clinical isolates expressing IMP-1 were cultured overnight and then diluted 100-fold in fresh LB medium. The bacterial suspension was dispensed into 96-well plates and incubated for 24 h in the presence of 2 μg/mL MEPM and various concentrations of DTPA-β-lactams. Bacterial growth was assessed by measuring turbidity at 655 nm. In addition, the concentrations of DTPA-β-lactams were fixed at 25 μM, and the bacteria were cultured in the presence of various concentrations of MEPM to determine the minimum inhibitory concentration (MIC) of MEPM.

### Fraction inhibitory concentration index (FICI)

The FICI was calculated using the following equation:


$$FICI=\left(\frac{{MIC}_{\text{DTPA-}\beta \text{-lactam combined}}}{{MIC}_{\text{DTPA-}\beta \text{-lactam alone}}}\right)+\left(\frac{{MIC}_{\text{meropenem combined}}}{{MIC}_{\text{meropenem alone}}}\right).$$

The concentration of the “MIC of DTPA-β-lactam” was set at 100 μM to determine the FIC index value. For the combination, the concentrations of DTPA-β-lactams and MEPM were fixed at 25 μM and 2 μg/mL, respectively. FICI values of ≤0.5 were set to indicate synergistic effects.

### Antibiotic susceptibility test

Antibiotic susceptibility testing was performed by using ready-state Dry Plates (DP-45) (Eiken Chemical Co., Ltd., Tokyo, Japan). Each plate contained 22 antimicrobial agents, including piperacillin, tazobactam/piperacillin, cefepime, ceftazidime, ceftazidime/dipicolinic acid (DPA), cefozopran, gentamicin, minocycline, doripenem, amikacin, levofloxacin, aztreonam, IPM, IPM/DPA, MEPM, MEPM/DPA, colistin, tobramycin, ciprofloxacin, sulfamethoxazole/trimethoprim, sulbactam/cefoperazone, and fosfomycin. Overnight cultures of KP1 were diluted 1,000-fold in Mueller-Hinton Broth (Becton Dickinson and Company, Franklin Lakes, NJ, USA). The suspension with 0.1 mL was added to each well and incubated for 20 h at 37°C. The MIC for each antibiotic was determined by visual inspection.

### Animal studies

Male 4-week-old ddY mice were purchased from Japan SLC Inc. (Shizuoka, Japan) and housed in the Center for Animal Resources and Development, Kumamoto University. Mice were divided into groups of six per cage for each experiment.

### Mouse infection model with *K. pneumoniae*

For the survival assay, mice were randomly divided into four groups (PBS/PBS, PBS/MEPM, DTPA/MEPM, and DTPA-ADCA/MEPM), with six mice in each group. Mice were intraperitoneally (i.p.) infected with KP-1 at the indicated dose. At 30 min post-infection, mice received a subcutaneous (s.c.) injection of either PBS, DTPA (60.6 mg/kg), or DTPA-ADCA (100 mg/kg). Subsequently, mice were subcutaneously administered either PBS or MEPM (60 mg/kg) at 60 min post-infection. Survival was monitored for 10 days following infection.

### Colony-forming units (CFU) assay

At 24 h post-infection, mice were euthanized, and blood samples were collected. In some experiments, at 48 h post-infection, blood, liver, and spleen samples were collected from PBS/PBS-treated or DTPA-ADCA/MEPM-treated groups. The liver was placed in PBS containing 1% sodium deoxycholate and mechanically disrupted using sterile scissors. The tissue homogenates were then ground thoroughly by using a Sterile Cell Strainer 70 μm (Thermo Fisher Scientific Inc., Waltham, MA, USA) to ensure complete lysis. The spleen was gently crushed using a bent needle. Blood samples were collected in tubes containing 4 mM EDTA to prevent coagulation. All samples were serially diluted in PBS and plated onto 1.5% LB agar plates (1.5% agar [Nissui Pharmaceutical Co. Ltd, Tokyo, Japan]) containing 100 μg/mL AMP. The plates were incubated at 37°C, and CFU were counted to assess bacterial load.

### Statistical analysis

All data are expressed as means ± standard deviation (SD). Data for each experiment were acquired from at least three independent experiments. Student’s *t*-tests were performed to assess differences between the two groups. For comparisons among multiple groups, one-way analysis of variance (ANOVA) was conducted, followed by Tukey’s multiple-comparison test. Survival curves constructed by using the Kaplan-Meier method were analyzed by means of log-rank (Mantel-Cox) test. Statistical significance was set at a *P* value of <0.05.

## RESULTS

### Syntheses of DTPA-β-lactams

**Fig 1:**
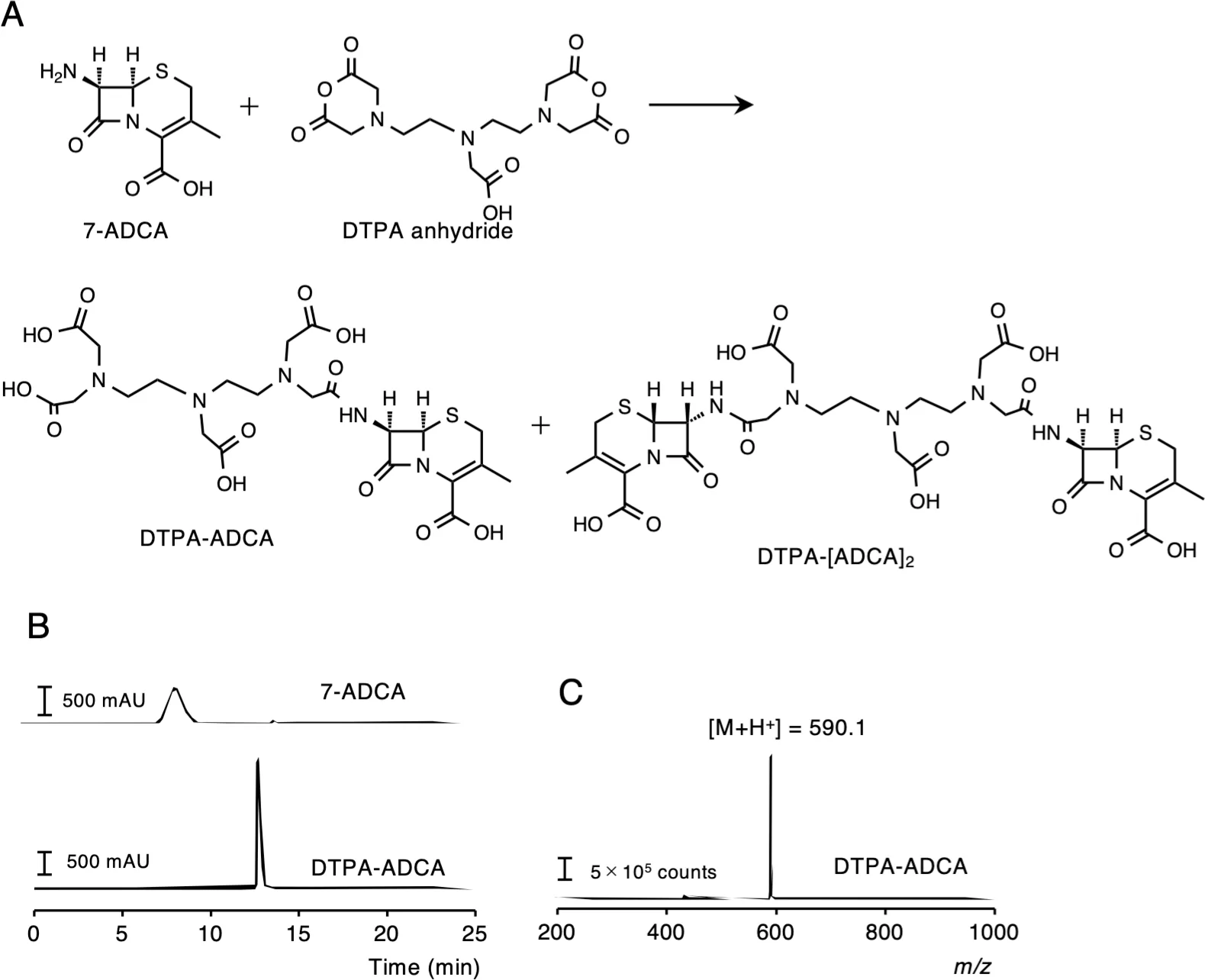
Synthesis and characterization of DTPA-ADCA. (**A**) Synthetic route for DTPA-ADCA. (**B**) HPLC profiles of 7-ADCA (upper panel) and DTPA-ADCA (lower panel). (**C**) Mass chromatogram of DTPA-ADCA.

**Fig 2:**
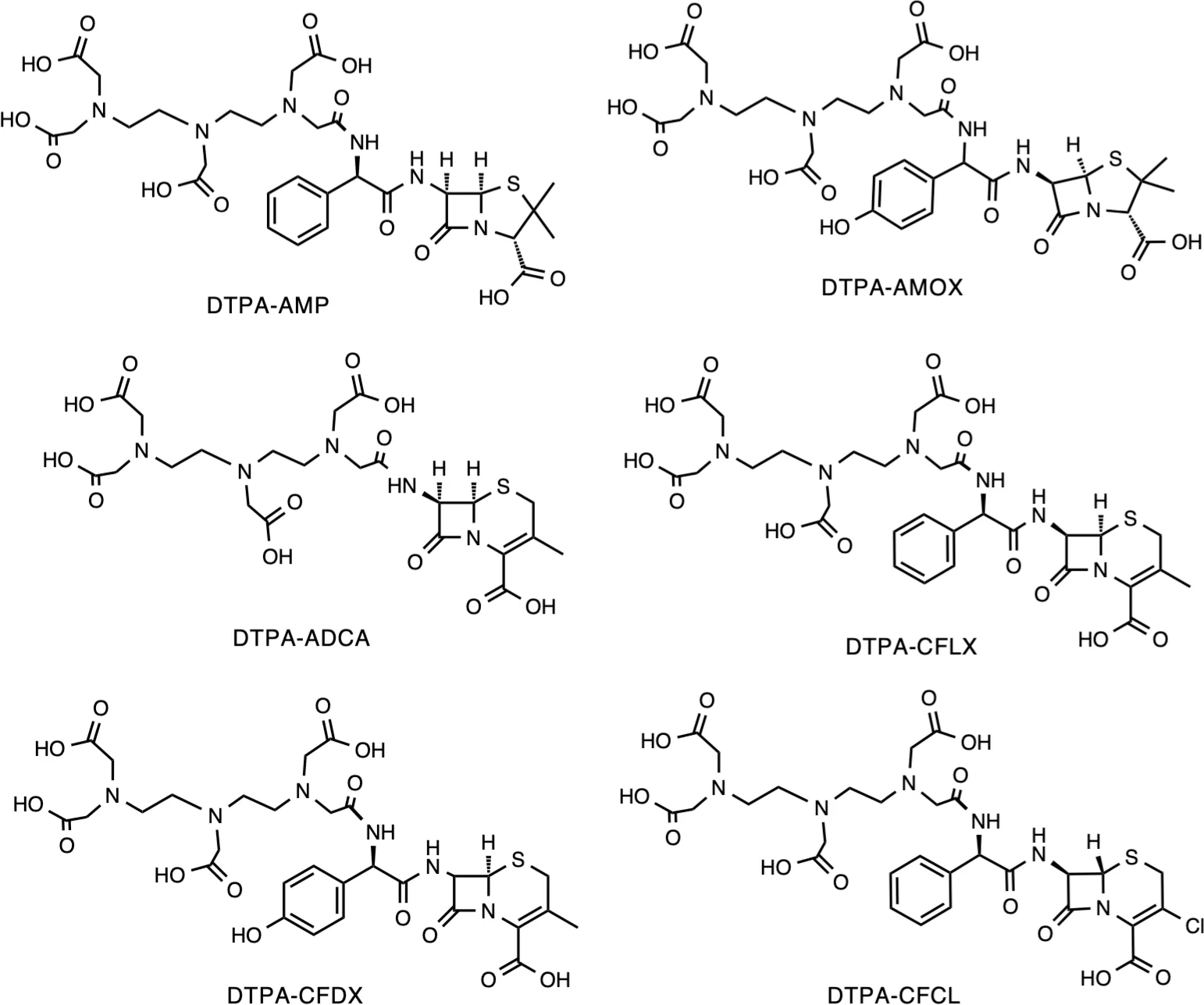
Chemical structures of DTPA-β-lactams synthesized in this study.

DTPA-β-lactams were synthesized by reacting DTPA anhydride with β-lactams that possess primary amino groups as reported previously (14). Figure 1 shows the synthesis of DTPA-ADCA as an example. As shown in Fig. 1A, the reaction between DTPA anhydride and ADCA forms DTPA-ADCA and DTPA-[ADCA]_2_ as main products. DTPA-ADCA was purified from the reaction mixture by solid-phase extraction and HPLC purification. By using this protocol, we could obtain highly purified DTPA-ADCA (Fig. 1B). Mass spectrometry analysis confirmed conjugation of the DTPA moiety to ADCA (Fig. 1C). In this study, we could successfully synthesize six DTPA-β-lactams having different β-lactam structures (Fig. 2; Fig. S1).

### Inhibitory activities of DTPA-β-lactams against MBL IMP-1 and VIM-2

**Fig 3:**
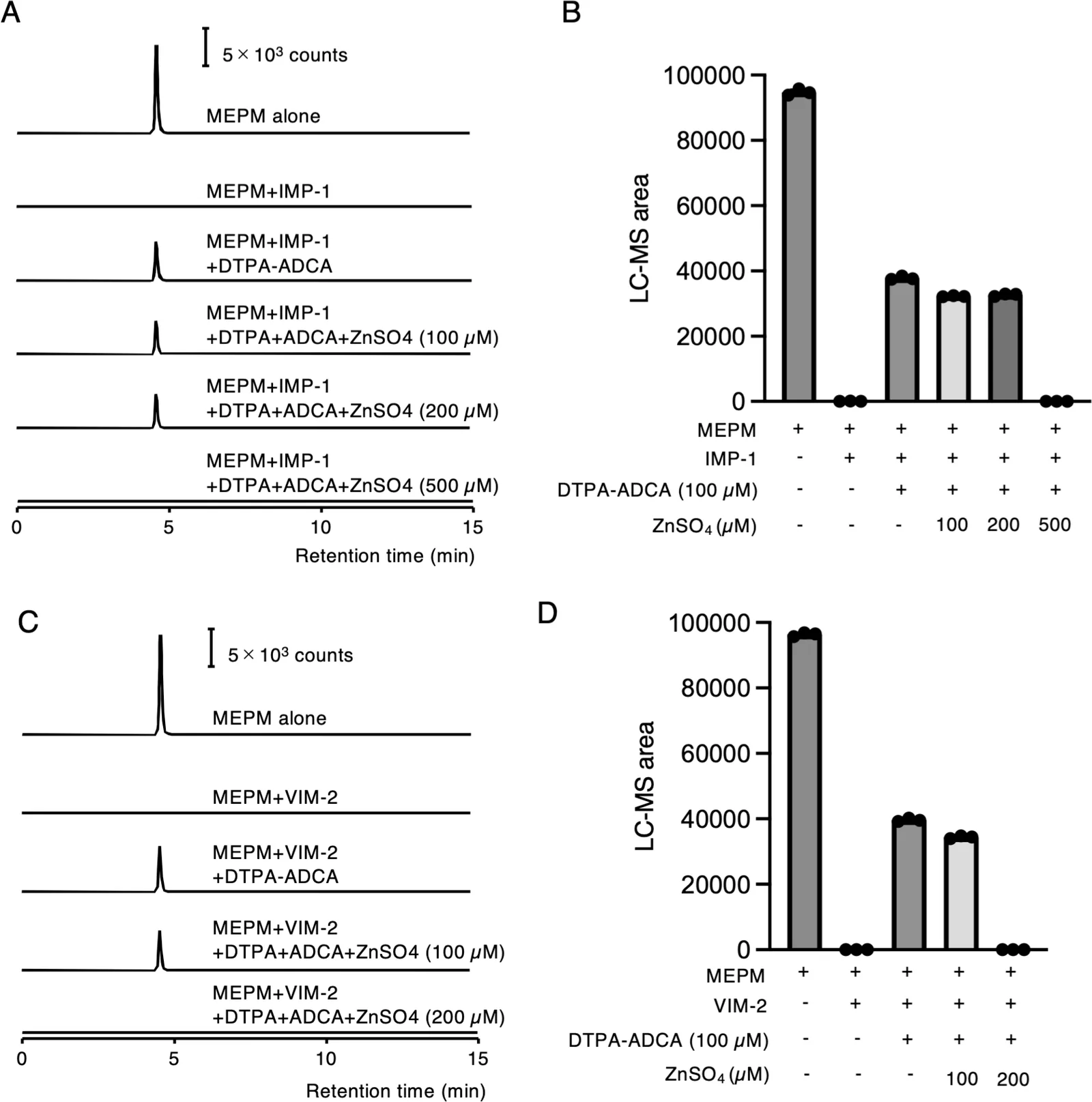
Effects of DTPA-ADCA on IMP-1– and VIM-2–mediated meropenem degradation. Meropenem (100 μM) was incubated in the presence or absence of 300 nM IMP-1 (**A and B**) or VIM-2 (**C and D**) at 37°C for 30 min in 50 mM HEPES buffer (pH 7.5) containing 200 mM NaCl. In some experiments, zinc sulfate was added to the reaction mixtures at the indicated concentrations. Meropenem levels in the reaction mixtures were quantified by LC–MS/MS. (**A and C**) Representative chromatograms. (**B and D**) Quantitative analysis of the data shown in panels **A** and **C**. Data are presented as the mean ± SD (*n* = 3).

**Fig 4:**
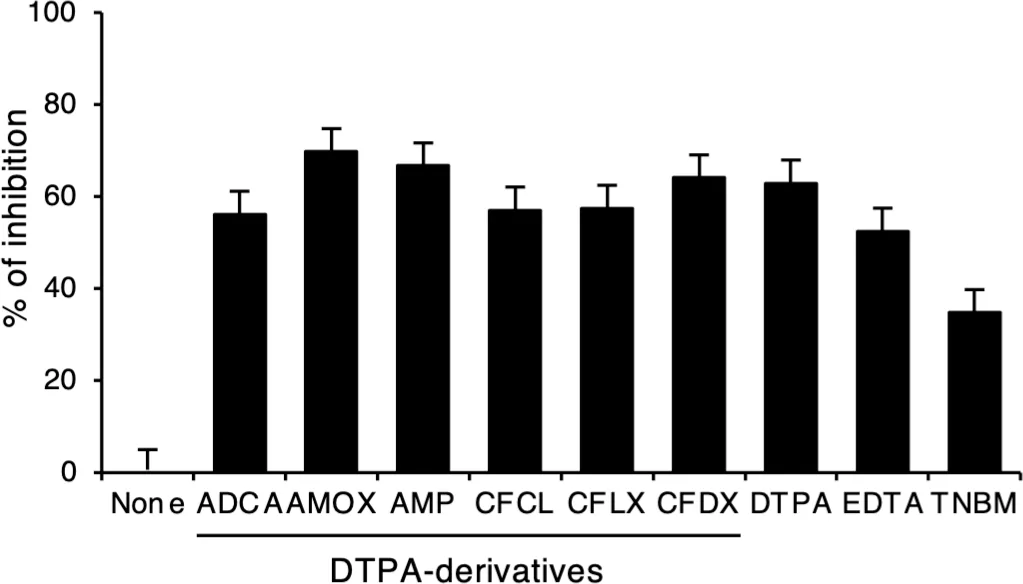
Inhibitory activities of DTPA-β-lactams against IMP-1. Meropenem (100 μM) was incubated with IMP-1 (100 nM) in the presence of the indicated compounds (100 μM) at 37°C for 30 min in 50 mM HEPES buffer (pH 7.5) containing 200 mM NaCl. The inhibitory effects of the test compounds were evaluated based on the remaining amount of meropenem. Data are presented as the mean ± SD (*n* = 3).

Inhibitory activities of DTPA-β-lactams against MBLs, including IMP-1 and VIM-2, were investigated by using recombinant IMP-1 and VIM-2. The His-tagged enzymes were expressed in *E. coli* and were purified by means of Ni-NTA agarose affinity chromatography. As shown in Fig. S2, recombinant IMP-1 and VIM-2 were effectively purified under the current condition. The enzyme reaction was examined by monitoring the degradation of substrate (MEPM) in the presence of recombinant enzymes with or without test compounds. As shown in Fig. 3, complete degradation of MEPM was observed in the presence of IMP-1 (Fig. 3A and B) and VIM-2 (Fig. 3C and D), suggesting that the recombinant enzymes maintained sufficient enzyme activities. Addition of DTPA-ADCA to the enzyme reaction mixtures inhibited MEPM degradation by approximately 50% compared with the condition in the absence of DTPA-ADCA (Fig. 3). Addition of ZnSO_4_ to the enzyme reaction mixtures canceled the inhibitory effects of DTPA-ADCA. The inhibition of IMP-1 by DTPA-ADCA was completely abolished in the presence of 500 μM ZnSO_4_, whereas the inhibition of VIM-2 was completely abolished in the presence of 200 μM ZnSO_4_. These results suggest that the inhibitory effects of DTPA-ADCA against MBLs are zinc-dependent. Inhibitory effects against IMP-1 were observed for all DTPA-β-lactams synthesized in this study. The inhibitory activities of these DTPA-β-lactams, evaluated based on MEPM degradation, were comparable to those of the chelators DTPA and EDTA. TNBM, used as a comparator, also showed a similar but slightly weaker inhibitory effect (Fig. 4).

Imipenemase inhibitory activities of DTPA-β-lactams against IMP-1 and VIM-2 were further analyzed by means of spectrophotometric IPM degradation assay (20). As shown in Fig. S3, simple chelators including EDTA and DTPA inhibited IMP-1 and VIM-2 in a dose-dependent manner. However, IC_50_ values of these compounds against IMP-1 were determined to be higher than 100 μM (Fig. S3; Table 1). DTPA-β-lactams including DTPA-AMOX, DTPA-ADCA, and DTPA-AMP showed stronger inhibition against IMP-1 than those by DTPA and EDTA. To further clarify the contribution of the β-lactam moiety, we evaluated the inhibitory activity of β-lactam alone against IMP-1. AMOX alone exhibited only weak inhibition, with an IC_50_ value greater than 100 µM, indicating a minimal effect on IMP-1 activity (Fig. S3A). In contrast, as mentioned above, DTPA alone inhibited IMP-1 activity by approximately 50% at 100 µM, whereas the DTPA–AMOX showed markedly enhanced inhibition, with an IC_50_ value of 28.8 µM. These results suggest that conjugation of the β-lactam moiety of DTPA significantly enhances inhibitory activity. In order to further confirm whether DTPA conjugation to β-lactam was essential for the enhanced inhibitory activity observed for DTPA-β-lactam, IMP-1 activity was determined in the presence of a simple mixture of ADCA and DTPA. As shown in Fig. S3 and S4, 100 μM DTPA inhibited IMP-1 activity by approximately 50%, and the addition of ADCA did not further enhance this inhibitory effect. In contrast, DTPA-ADCA at 100 μM inhibited IMP-1 to 38%. IMP-1 inhibition determined by imipenem degradation by DTPA-conjugated cephems, including DTPA-CFCL, DTPA-CFDX, and DTPA-CFLX, was not determined due to interference of the absorbance derived from those compounds. Under these conditions, the IC_50_ value of TNBM to IMP-1 was approximately 5 μM. This indicates that DTPA-β-lactam has 5- to 17-fold lower MBL inhibitory potency than TNBM to IMP-1.

**TABLE 1:** IC_50_ values of test compounds on recombinant IMP-1 and VIM-2^*a*^^,^^*b*^

Reagents	IC_50_ (μM)	
	IMP-1	VIM-2
DTPA	>100	0.342
EDTA	100	0.343
DTPA-AMOX	26.75	0.009
DTPA-ADCA	38.80	0.134
DTPA-AMP	84.8	0.343
ADCA/DTPA	>100	n.d.
Taniborbactam	5.24	0.557

^*a*^ Enzyme reactions were carried out at 30°C in 50 mM HEPES-NaOH buffer (pH 7.5) containing 200 mM NaCl, using 100 nM enzymes in the presence of 200 μM imipenem. IC_50_ values were determined from Fig. S3.

^*b*^ n.d., not determined.

The inhibitory effects of DTPA-β-lactams against VIM-2 were also evaluated. VIM-2 was more susceptible to chelating compounds than IMP-1. The IC_50_ values of the chelators DTPA and EDTA against VIM-2 were approximately 0.34 μM. TNBM showed a slightly weaker inhibitory effect than DTPA and EDTA, with an IC_50_ value of approximately 0.5 μM. Among the DTPA-β-lactams synthesized in this study, DTPA-AMOX and DTPA-ADCA exhibited stronger inhibitory effects than DTPA and EDTA, with IC_50_ values of 0.009 and 0.134 μM, respectively (Table 1).

**Fig 5:**
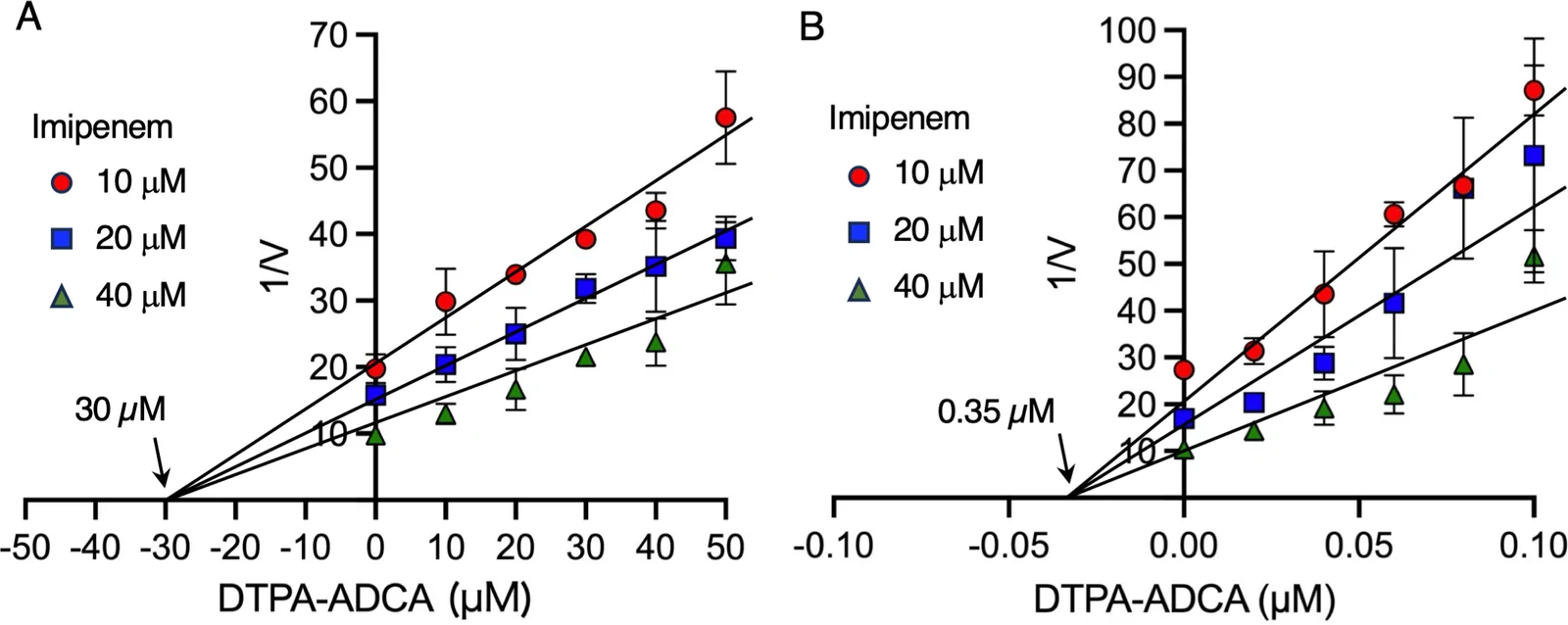
Dixon plots for inhibition of IMP-1 and VIM-2 by DTPA-ADCA. Imipenem was used as the substrate at the indicated concentrations and incubated with 100 nM IMP-1 or VIM-2 in the presence of the indicated concentrations of DTPA-ADCA. The *y*-axis represents the reciprocal of the decrease in absorbance at 298 nm, which reflects imipenem hydrolysis. Data were obtained from at least two independent experiments. The inhibition constants (*K*_i_) of DTPA-ADCA for IMP-1 (**A**) and VIM-2 (**B**) were determined to be 30 and 0.35 μM, respectively.

The inhibitory constants (*K*_i_) of DTPA-ADCA against MBLs were determined using Dixon plots. As shown in Fig. 5, the *K*_i_ values of DTPA-ADCA were calculated to be 30 μM for IMP-1 and 0.35 μM for VIM-2.

**Fig 6:**
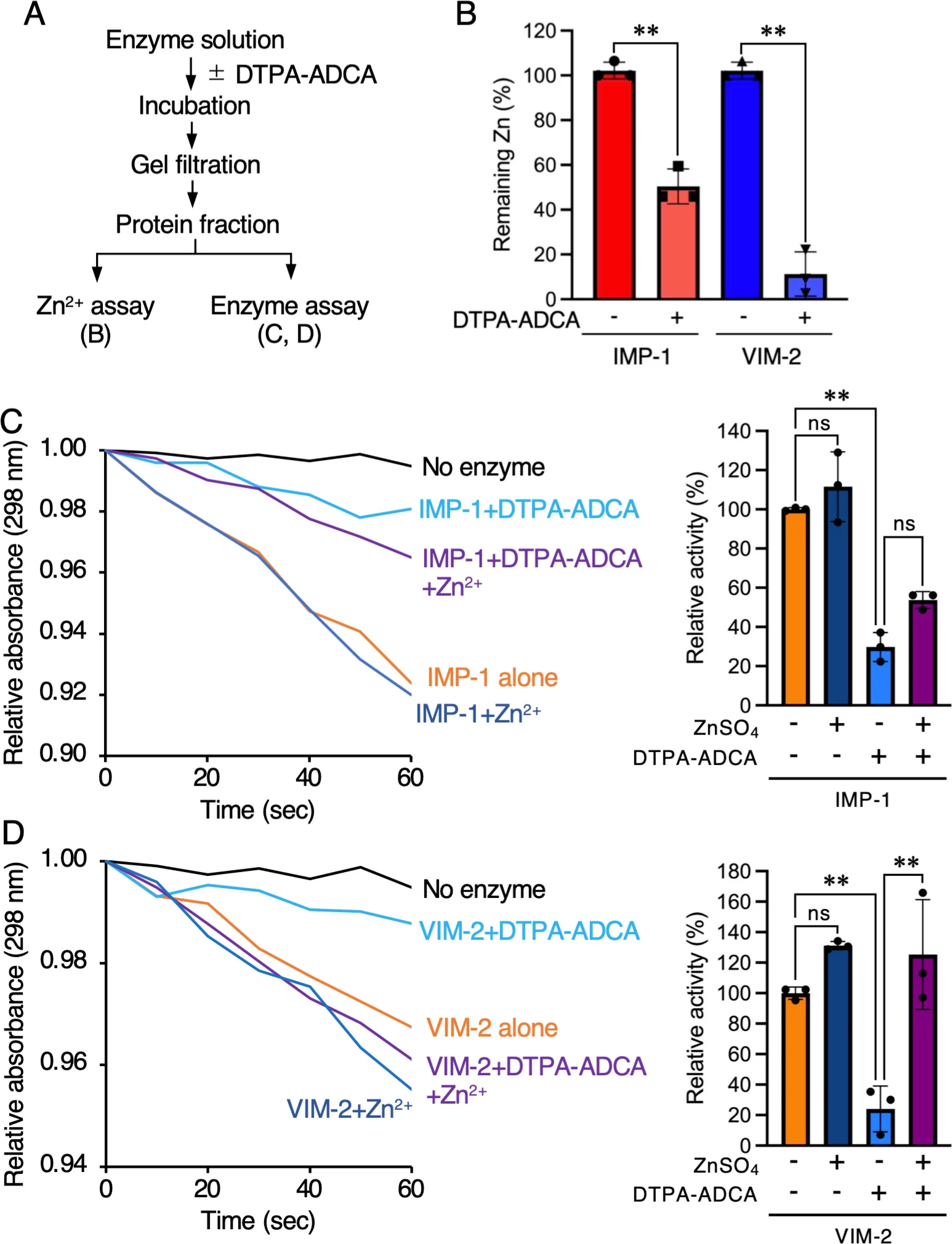
Effects of DTPA-ADCA treatment on the enzyme activity and zinc content of IMP-1 and VIM-2. (**A**) Experimental protocol. IMP-1 or VIM-2 (10 μM) was incubated in the presence or absence of DTPA-ADCA (100 μM) at 37°C for 30 min in 50 mM HEPES buffer (pH 7.5), followed by gel filtration. High-molecular-weight fractions containing the enzymes were collected and subjected to zinc quantification (**B**) and enzyme activity assays (**C and D**). (**B**) Zinc contents of IMP-1 and VIM-2 with or without DTPA-ADCA treatment. Zinc levels were determined using a Metalloassay kit. Values are expressed relative to samples without DTPA-ADCA treatment, which were set to 100%. (**C and D**) Enzyme activities of IMP-1 (**C**) and VIM-2 (**D**). Enzyme activity was determined based on imipenem hydrolysis, monitored by the decrease in absorbance at 298 nm. The left panels show representative absorbance profiles. Enzyme activity is expressed relative to the enzyme-alone condition, which was set to 100%. To evaluate the effect of zinc supplementation, 100 μM zinc sulfate was added to the gel-filtered samples prior to the activity assay. Quantitative data are shown in the right panels. Data are presented as the mean ± SD (*n* = 3). Statistical analyses were performed using Student’s *t*-test (**B**) and one-way ANOVA followed by Tukey’s post hoc multiple-comparison test (**C and D**). **, *P* < 0.01; ns, not significant.

As shown above, the inhibitory effects of DTPA-ADCA on IMP-1 and VIM-2 were abolished by the addition of zinc sulfate to the reaction mixtures. We therefore investigated the zinc dependency of DTPA-ADCA inhibition in greater detail. After incubation of the MBLs with DTPA-ADCA, low-molecular-weight fractions were removed by gel filtration, and the amount of zinc bound to the protein fraction was quantified. In addition, the enzymatic activities of the treated enzymes and their recovery following zinc supplementation were evaluated (Fig. 6A).

Quantification of the zinc remaining in the MBLs after treatment with DTPA-ADCA showed that the zinc content of IMP-1 was reduced to approximately 50% of the original level (Fig. 6B), indicating that the remaining 50% of zinc was retained by IMP-1. This finding suggests that DTPA-ADCA remained bound to IMP-1 and co-eluted with the enzyme during gel filtration, thereby retaining a portion of the zinc associated with IMP-1. In contrast, the zinc content of VIM-2 decreased to approximately 10% of the original level (Fig. 6B), indicating that DTPA-ADCA extracted zinc from VIM-2 and that the resulting zinc–DTPA-ADCA complex was removed during gel filtration.

Enzymatic activity assays showed that the activities of both enzymes remained markedly reduced even after gel filtration (Fig. 6C and D). In the case of IMP-1, supplementation with zinc sulfate resulted in only partial recovery of enzymatic activity. Consistent with the results described above, this finding suggests that DTPA-ADCA binds strongly to IMP-1, and that the amount of zinc added was insufficient to fully restore enzyme activity. In contrast, the activity of VIM-2 was almost completely restored by the addition of zinc sulfate. Taken together, these results suggest that DTPA-ADCA inhibits VIM-2 by extracting zinc from the enzyme in an effectively irreversible manner.

### Antimicrobial activities of DTPA-β-lactams on IMP-1-expressing Enterobacterales

**Fig 7:**
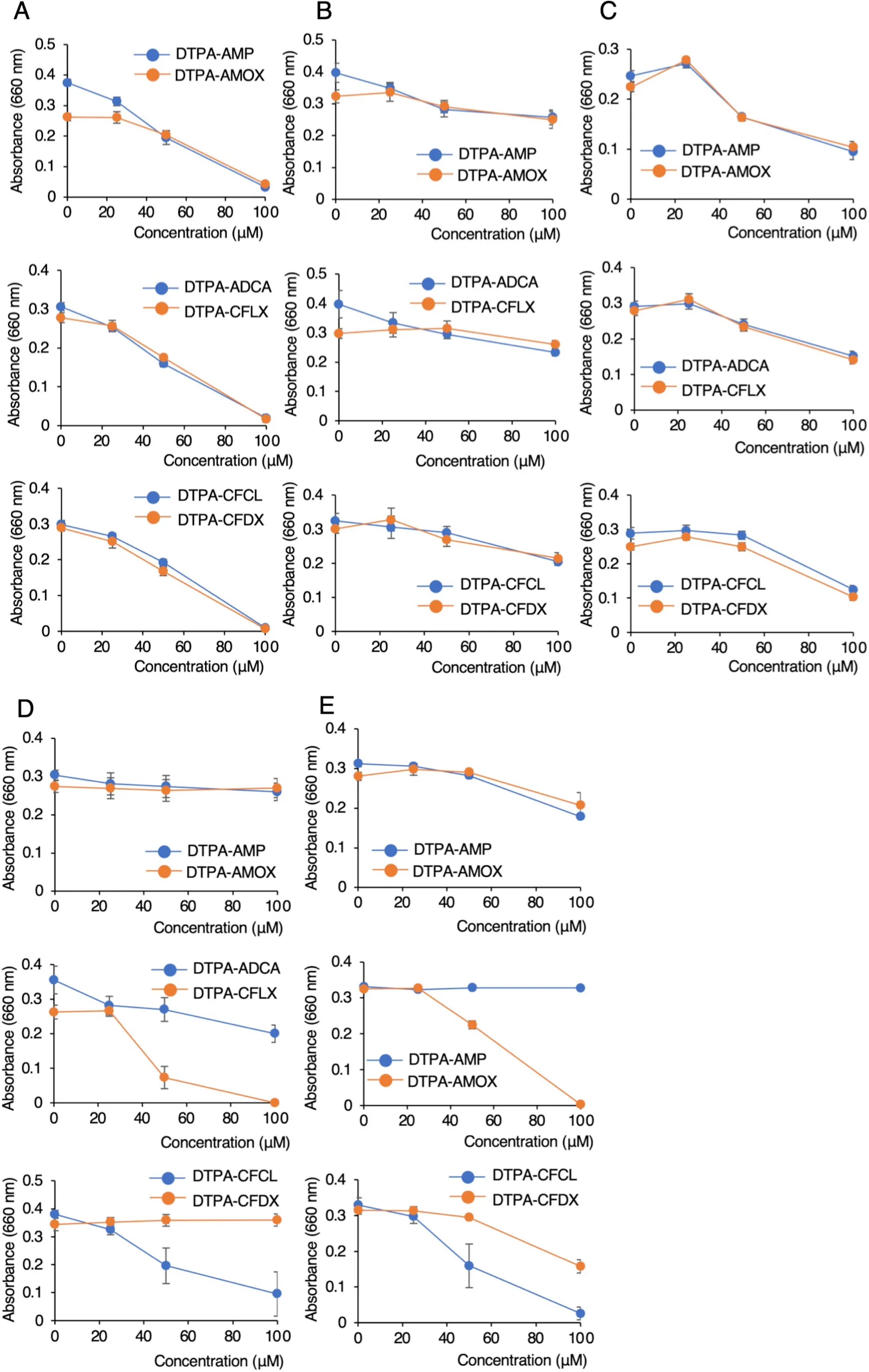
Antibacterial effects of DTPA-β-lactams. Clinical isolates were cultured in LB medium containing the indicated concentrations of DTPA-β-lactams. The bacterial strains tested were (**A**) *C. amalonaticus*, (**B**) *E. coli*, (**C**) *C. freundii*, (**D**) KP1, and (**E**) KP2. The DTPA-β-lactams used were as follows: top panels, DTPA-AMP and DTPA-AMOX; middle panels, DTPA-ADCA and DTPA-CFLX; and bottom panels, DTPA-CFCL and DTPA-CFDX. Data are presented as the mean ± SD (*n* = 3).

We investigated the effects of DTPA-β-lactams on carbapenem susceptibility of bacteria expressing IMP-1. All clinical isolates, including KP1, KP2, *E. coli*, *C. freundii*, and *C. amalonaticus*, expressed IMP-1 (Fig. S5) and were highly resistant to MEPM. The MIC values of MEPM against these bacteria ranged from 8 to 16 μg/mL (Fig. S6; Table 2). We also examined the antibacterial activities of the synthesized DTPA-β-lactams themselves. Although slight differences were observed among bacterial strains, the DTPA-β-lactams showed only weak direct antibacterial activity, with MIC values of 100 μM or higher (Fig. 7; Tables 2 and 3). None of the DTPA-β-lactams affected bacterial growth at a concentration of 25 μM (Fig. 7).

**TABLE 2:** FICI values of DTPA-ADCA and meropenem determined against gram-negative clinical isolates

Strains	MIC of DTPA-ADCA (μM)		MIC_DTPA-ADCA with MEPM_/ MIC_DTPA-ADCA_	MIC of MEPM (μg/mL)		MIC_MEPM with DTPA-ADCA_/ MIC_MEPM_	FICI^*d*^
	Alone^*a*^	With MEPM^*b*^		Alone	With DTPA-ADCA^*c*^		
KP1	100	25	0.25	16	1	0.0625	0.3125
KP2	100	25	0.25	16	0.5	0.03125	0.28125
*E. coli*	100	15	0.15	10	0.5	0.05	0.2
*C. amalonaticus*	100	15	0.15	8	0.125	0.0156	0.1656
*C. freundii*	100	20	0.2	8	1	0.125	0.325

^*a*^ The concentration of the “MIC of DTPA-ADCA alone” was set at 100 μM to determine the FIC index value.

^*b*^ Meropenem concentration was fixed at 2 μg/mL.

^*c*^ DTPA-ADCA concentration was fixed at 25 μM.

^*d*^ FICI = (MIC_DTPA-ADCA with MEPM_/MIC_DTPA-ADCA_) + (MIC_MEPM with DTPA-ADCA_/MIC_MEPM_).

**TABLE 3:** FICI values of DTPA-β-lactams and meropenem determined against KP1

Compounds	MIC of DTPA-β-lactam (μM)		MIC_DTPA-β-lactam with MEPM_/ MIC_DTPA-β-lactam_	MIC of MEPM (μg/mL)		MIC_MEPM with DTPA-β-lactam_/ MIC_MEPM_	FICI^*e*^
	Alone^*a*^	With MEPM^*b*^		Alone	With DTPA-β-lactam^*c*^		
DTPA-AMOX	100	25	0.25	16	0.06	0.0037	0.2537
DTPA-AMP	100	25	0.25	16	0.06	0.0037	0.2537
DTPA-ADCA	100	25	0.25	16	1	0.0625	0.3125
DTPA-CFLX	100	25	0.25	16	0.06	0.0037	0.2537
DTPA-CFDX	100	25	0.25	16	0.06	0.0037	0.2537
DTPA-CFCL	n.d.^*d*^	n.d.	–^*f*^	n.d.	n.d.	–	–

^*a*^ The concentration of the “MIC of DTPA-β-lactam” was set at 100 μM to determine the FIC index value.

^*b*^ Meropenem concentration was fixed at 2 μg/mL.

^*c*^ DTPA-β-lactam concentration was fixed at 25 μM.

^*d*^ n.d, not determined.

^*e*^ FICI = (MIC_DTPA-β-lactam with MEPM_/MIC_DTPA-β-lactam_) + (MIC_MEPM with DTPA-β-lactam_/MIC_MEPM_).

^*f*^ “–” indicates not applicable.

**Fig 8:**
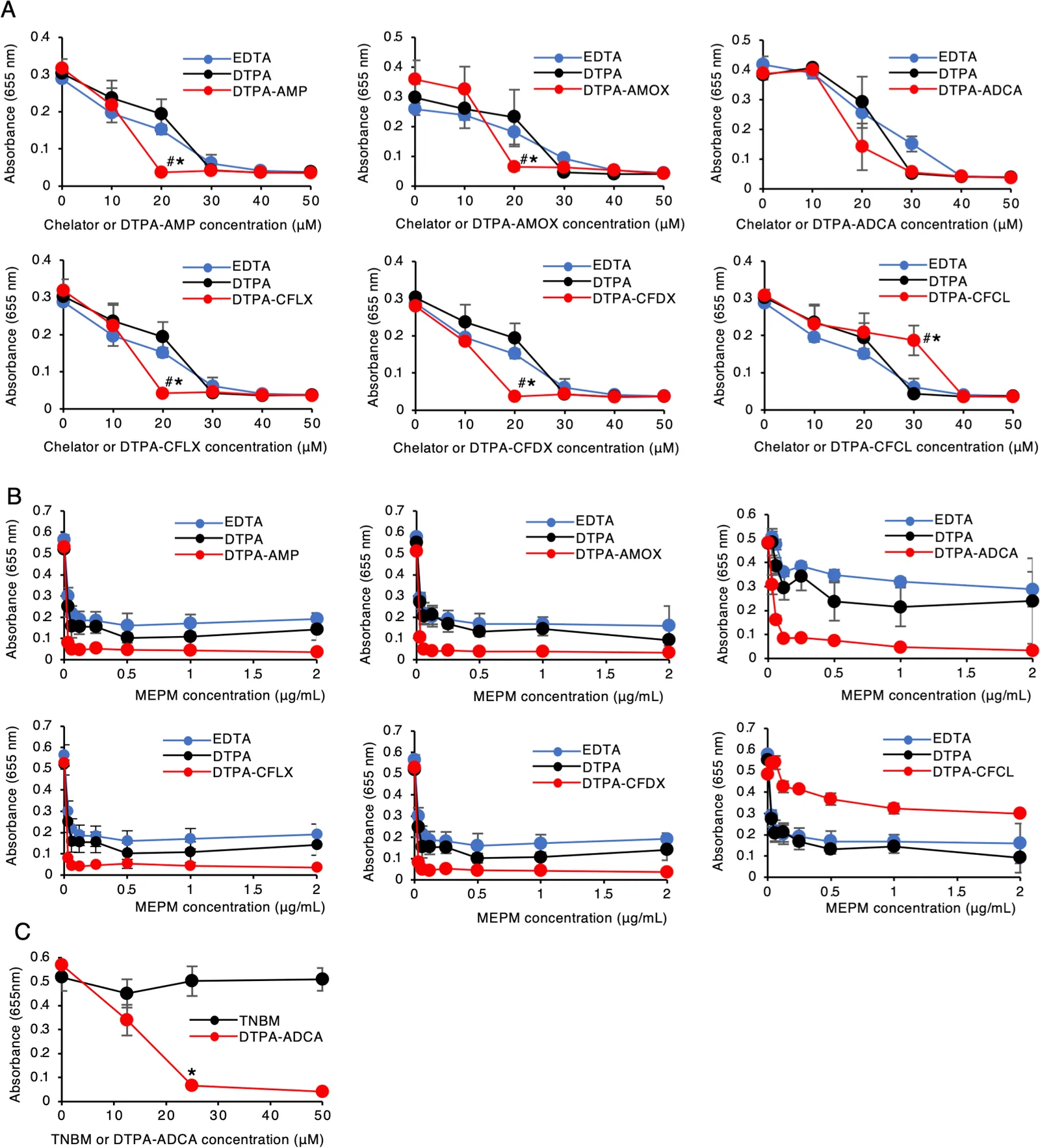
Effects of DTPA-β-lactams on the meropenem sensitivity of KP1. (**A**) KP1 cells were cultured in LB medium containing 2 μg/mL meropenem (MEPM) in combination with EDTA, DTPA, or DTPA-β-lactams. (**B**) KP1 cells were cultured in LB medium containing 25 μM EDTA, DTPA, or DTPA-β-lactams together with the indicated concentrations of MEPM. (**C**) Sensitivity of KP1 to DTPA-ADCA and TNBM in the presence of 0.5 μg/mL MEPM. Bacterial growth was evaluated by measuring turbidity at 655 nm. Data are presented as the mean ± SD (*n* = 3). Statistical significance was determined using an unpaired two-tailed Student’s *t*-test. #, EDTA vs DTPA-ADCA, *P* < 0.05; *, DTPA vs DTPA-ADCA, *P* < 0.05; ns, not significant. In panel **C**, *P* < 0.05 for TNBM vs DTPA-ADCA.

Next, we investigated the combinational effects of DTPA-β-lactams and MEPM. Figure 8A shows the results obtained when the concentration of MEPM was fixed at 2 μg/mL and the concentrations of DTPA-β-lactams were varied, while Fig. 8B shows the effects on KP1 when the concentration of DTPA-β-lactams was fixed at 25 μM. For comparison, the results obtained using DTPA and EDTA are also shown. All DTPA-β-lactams except DTPA-CFCL enhanced the antibacterial activity of MEPM against KP1. Among them, DTPA-AMP, DTPA-AMOX, DTPA-CFLX, and DTPA-CFDX exhibited significantly greater potentiating effects on MEPM activity than either DTPA or EDTA. Although the effect of 20 μM DTPA-ADCA did not differ significantly from those of DTPA and EDTA, it showed a tendency toward greater growth suppression. In contrast, DTPA-CFCL exhibited a weaker potentiating effect on MEPM activity than either DTPA or EDTA (Fig. 8A). Similarly, all DTPA-β-lactams except DTPA-CFCL markedly reduced the MIC of MEPM against KP1 when used in combination with MEPM (Fig. 8B). In contrast, TNBM showed no antibacterial effect against KP1 even in combination with MEPM (Fig. 8C).

**Fig 9:**
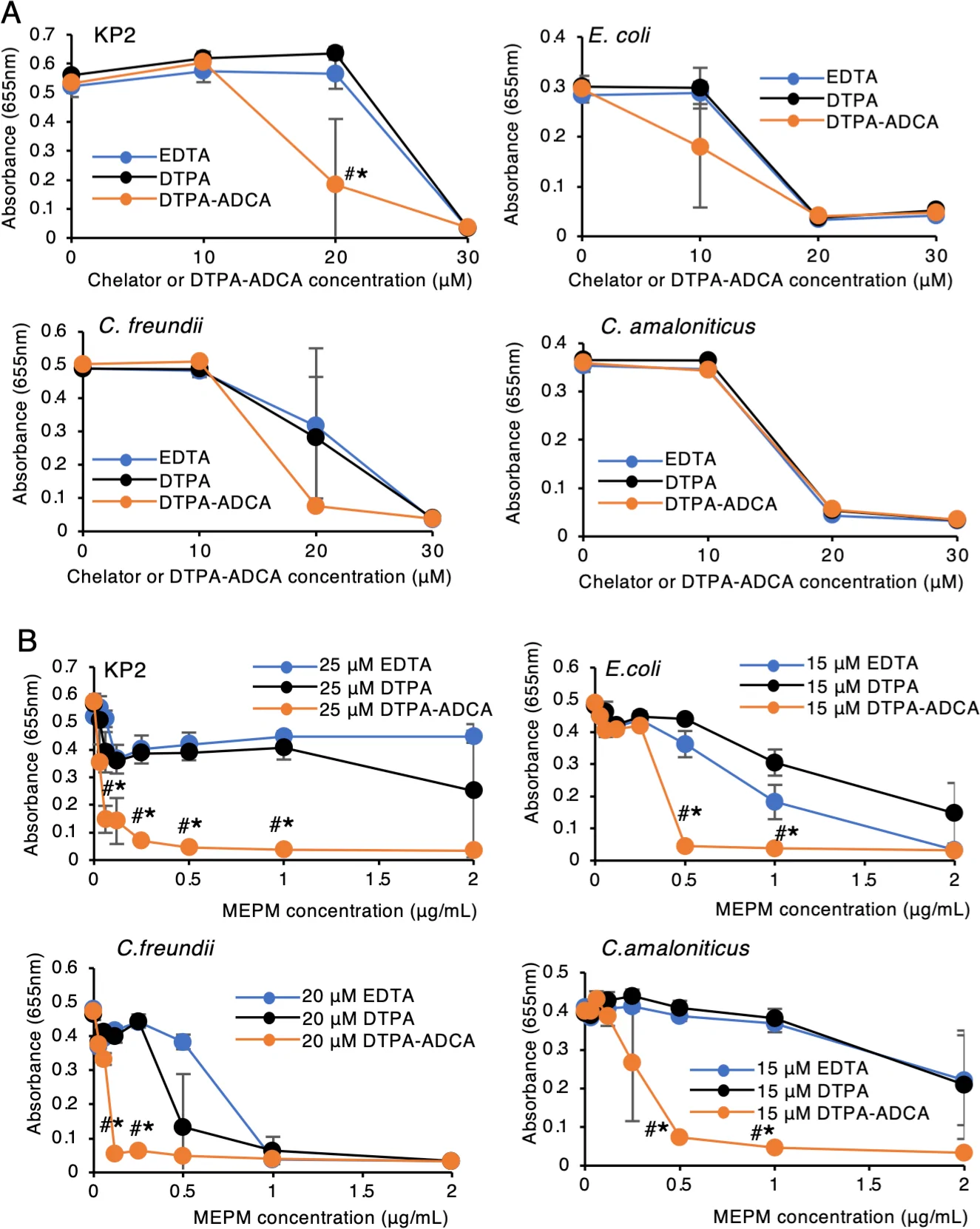
Antibacterial activity of the combination of meropenem and DTPA-ADCA against IMP-1-expressing bacterial strains. (**A**) Clinical isolates were cultured in LB medium containing 2 μg/mL meropenem (MEPM) together with various concentrations of the indicated additives. (**B**) The dose-dependent effects of MEPM on clinical isolates were examined in the presence of fixed concentrations of EDTA, DTPA, or DTPA-ADCA, as indicated in the figures. Bacterial growth was assessed by measuring absorbance at 655 nm. Data are presented as the mean ± SD (*n* = 3). Statistical significance was determined using an unpaired two-tailed Student’s *t*-test. #, EDTA vs DTPA-β-lactams, *P* < 0.05; *, DTPA vs DTPA-β-lactams, *P* < 0.05; ns, not significant.

The results obtained for bacterial strains other than KP1 using DTPA-ADCA are shown in Fig. 9. As observed for KP1, DTPA-ADCA markedly improved MEPM susceptibility when used in combination with MEPM. We therefore investigated whether the combination of DTPA-β-lactams and MEPM exhibited synergistic effects. Table 2 shows the fractional inhibitory concentration index (FICI) values for the combination of DTPA-ADCA and MEPM. For all bacterial strains examined in this study, the FICI values of DTPA-ADCA and MEPM were 0.5 or lower, indicating synergistic interactions between the two compounds. In addition, analysis of the combinations of DTPA-β-lactams and MEPM against KP1 demonstrated that five compounds, excluding DTPA-CFCL, also showed FICI values of 0.5 or lower, confirming synergistic effects with MEPM (Table 3).

The effects of DTPA-ADCA on the antibiotic susceptibility of KP1 were further investigated for diverse types of antibiotics. As summarized in Table 4, co-treatment with DTPA-ADCA enhanced the antibiotic susceptibilities of KP1 against seven out of nine β-lactam antibiotics. Particularly, MIC values of KP1 against IPM and MEPM were drastically decreased in the presence of DTPA-ADCA. On the other hand, MIC values for non-β-lactam antibiotics were not affected in the presence of DTPA-ADCA. These data clearly indicated that DTPA-ADCA potently acted as an IMP-1 inhibitor to improve the susceptibility of IMP-1-expressing bacteria against β-lactam antibiotics, including carbapenems.

**TABLE 4:** The MIC (μg/mL) of several types of antibiotics in the absence or presence of DTPA-ADCA against KP1^*a*^

DTPA-ADCA	PIPC	TAZ/PIPC	CFPM	CAZ	CZOP	AZT	IPM	MEPM	DRPM	S/C
None	>64	>4/64	>16	>32	>16	>16	>16	>16	>8	>32/32
50 μM	>64	>4/64	>16	8	16	>16	2	2	4	8/8
100 μM	>64	>4/64	8	4	4	>16	1	2	4	8/8

DTPA-ADCA	GM	MINO	AMK	LVFX	CL	FOM	TOB	CPFX	ST
None	>8	>8	>32	>4	>4	>128	>8	>2	>38/2
50 μM	>8	>8	>32	>4	4	>128	>8	>2	>38/2
100 μM	>8	>8	>32	>4	>4	>128	>8	>2	>38/2

^*a*^ Upper table; piperacillin (PIPC), tazobactam (TAZ)/PIPC, cefepime (CFPM), ceftazidime (CAZ), cefazopran (CZOP), aztreonam (AZT), imipenem (IPM), meropenem (MEPM), and doripenem (DRPM). Lower table; gentamycin (GM), Minocycline (MINO), amikacin (AMK), levofloxacin (LVFX), colistin (CL), tobramycin (TOB), ciprofloxacin (CPFX), sulfamethoxazole/trimethoprim (ST), sulbactam (S)/cefoperazone (C), and Fosfomycin (FOM).

It has been reported that indole-2-carboxylates act as Type II inhibitors for MBL by mimicking the binding mode of intact β-lactams (17). We synthesized DTPA-conjugated tryptophan (DTPA-tryptophan) as an indole-containing chelate compound and examined its effect on the carbapenem sensitivity of KP1. As shown in Fig. S7, DTPA-tryptophan treatment resensitized KP1 against MEPM almost equivalently to that caused by DTPA-β-lactams.

### Therapeutic effects of DTPA-ADCA/MEPM combination on lethal bacterial infection

**Fig 10:**
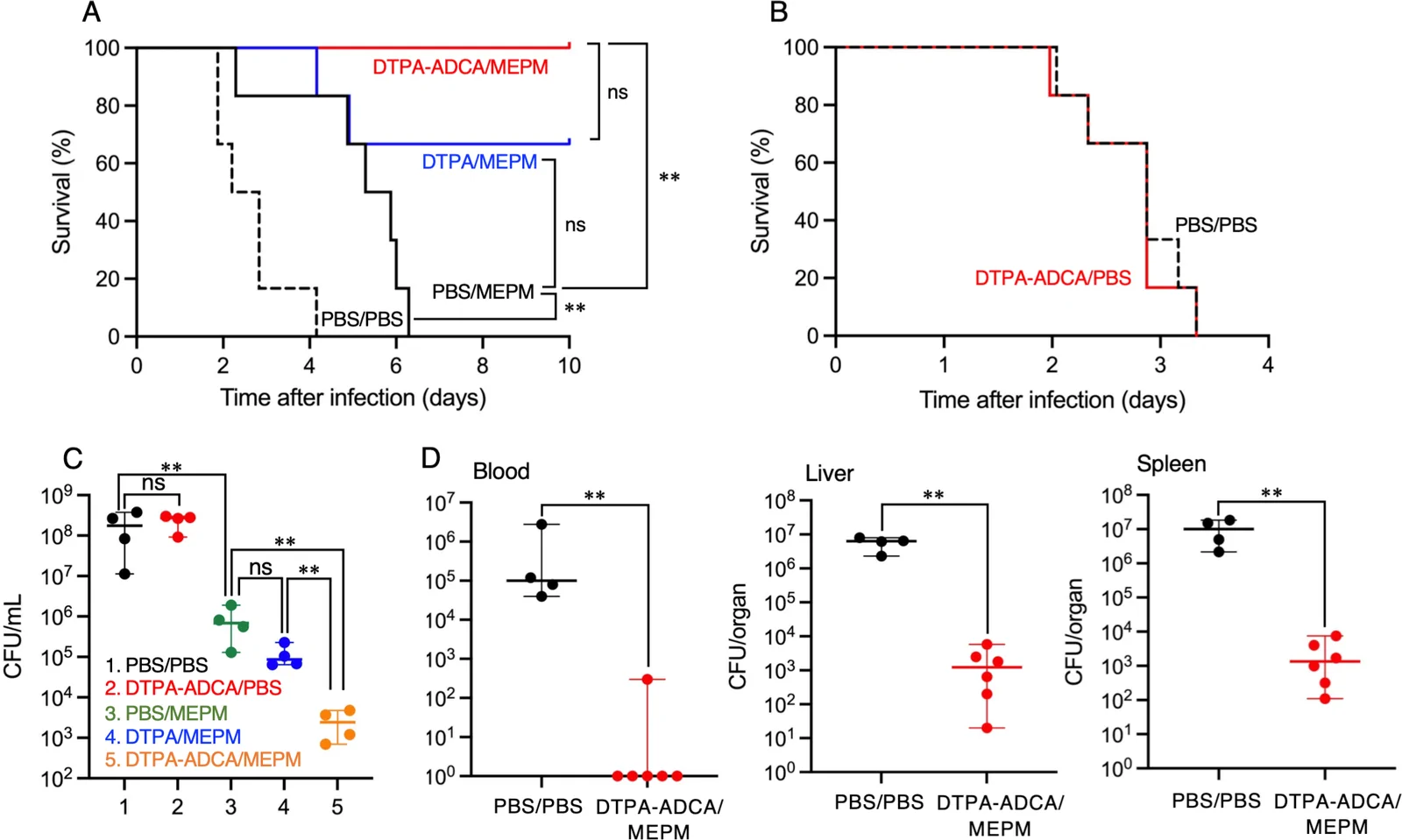
Therapeutic effects of the combination of DTPA-ADCA and meropenem in a mouse model of *K. pneumoniae* infection. (**A and B**) Survival of mice following KP1 infection. Four-week-old male ddY mice were intraperitoneally inoculated with 5 × 10^3^ CFU of KP1. Thirty minutes after infection, mice received subcutaneous administration of PBS, DTPA (60.6 mg/kg), or DTPA-ADCA (100 mg/kg). Thirty minutes later, PBS or meropenem (60 mg/kg) was administered subcutaneously. Survival curves were generated using the Kaplan-Meier method and analyzed by the log-rank (Mantel-Cox) test, with significance set at *P* < 0.05. (**C**) Changes in bacterial counts in the blood following each treatment. Mice were infected and treated as described in panels **A** and **B**, and blood samples were collected 24 h post-infection to determine KP1 bacterial loads. (**D**) Bacterial loads in the blood, liver, and spleen of KP1-infected mice following combination treatment with DTPA-ADCA and meropenem. Blood and organs were collected at 48 h post-infection, and bacterial counts were determined. The numbers of mice used were as follows: *n* = 6 for (**A and B**); *n* = 4 for (**C**); and for (**D**), *n* = 4 for the untreated PBS/PBS group and *n* = 6 for the DTPA-ADCA plus meropenem group. Statistical analyses were performed using one-way ANOVA followed by Tukey’s multiple-comparison test for (**C**) and Student’s *t*-test for (**D**). **, *P* < 0.01; ns, not significant.

We finally investigated the therapeutic effects of the DTPA-ADCA/MEPM combination against infections caused by IMP-1-expressing bacteria using a mouse model. We used KP1 as a representative IMP-1-expressing clinical isolate. When mice were intraperitoneally infected with KP1 at an inoculum of 5 × 10^3^ CFU/head, all mice died within approximately 3 days after infection (Fig. S8). Using this infection model, we evaluated the therapeutic effects of DTPA-ADCA alone, MEPM alone, and the combination of DTPA-ADCA and MEPM. As a treatment protocol, DTPA-ADCA or PBS as a control was subcutaneously administered 30 min after KP1 infection. An additional 30 min later, MEPM or PBS was subcutaneously administered. As shown in Fig. 10A, all mice in the untreated PBS/PBS group died within approximately 4 days after KP1 infection. In the MEPM monotherapy group, the time to death was significantly prolonged by approximately 2 days compared with the untreated PBS/PBS group; however, all mice eventually died. In addition, all mice in the DTPA-ADCA monotherapy group died with a time course almost identical to that of the untreated PBS/PBS group (Fig. 10B). In contrast, all mice in the DTPA-ADCA plus MEPM combination group survived. This combination therapy showed a significantly greater therapeutic effect than MEPM monotherapy. In the DTPA plus MEPM combination group, four out of six mice survived; however, no significant difference was observed compared with the MEPM monotherapy group.

We next examined whether the combination of DTPA-ADCA and MEPM reduced the bacterial burden in mice. As shown in Fig. 10C, approximately 10^8^ CFU/mL of KP1 was detected in the blood 1 day after infection in the untreated PBS/PBS group, indicating progression of septicemia. In the DTPA-ADCA monotherapy group, no significant change in blood bacterial counts was observed compared with the PBS/PBS group, consistent with our *in vitro* findings that DTPA-ADCA alone did not inhibit the growth of KP1 (Fig. 7B). Treatment with MEPM alone significantly reduced bacterial counts in the blood compared with the PBS/PBS group, which likely contributed to the prolonged survival observed in this group (Fig. 10A). However, complete bacterial clearance was not achieved under this treatment regimen. The combination of DTPA and MEPM did not significantly reduce blood bacterial counts compared with MEPM monotherapy. In contrast, the combination of DTPA-ADCA and MEPM resulted in a significantly greater reduction in blood bacterial counts than either MEPM monotherapy or the combination of DTPA and MEPM. We further evaluated bacterial burdens in the blood, liver, and spleen at 48 h after infection (Fig. 10D). In the DTPA-ADCA and MEPM-treated group, bacterial counts in the blood were below the detection limit in five of six mice, indicating near-complete eradication of bacteremia. Furthermore, viable bacterial counts in both the liver and spleen were significantly reduced compared with those in the untreated PBS/PBS group. These results demonstrate that the combination of DTPA-ADCA and MEPM markedly reduces the systemic bacterial burden in mice infected with MBL-producing bacteria.

## DISCUSSION

In this study, we demonstrated that DTPA-β-lactams exhibit potent inhibitory activity against MBLs, including IMP-1 and VIM-2. DTPA-β-lactams showed greater inhibitory activity against IMP-1 and VIM-2 than simple chelating agents such as DTPA and EDTA. The lack of comparable inhibitory activity when DTPA and β-lactam were simply mixed suggests that the spatial proximity between the chelating moiety and the β-lactam structure is crucial for effective MBL inhibition. It has been reported that, during the hydrolysis of β-lactam structure by MBL, the C-2 carboxylate of the bicyclic β-lactam interacts electrostatically with positively charged amino acid residues in MBL—such as K224 in IMP-1—thereby enhancing the binding affinity (21). A similar mechanism may apply to the DTPA-β-lactams, where the C-2 carboxylate of the bicyclic β-lactams enhances interaction with MBL, resulting in more potent inhibition than chelators alone.

The DTPA-β-lactams synthesized in this study exhibited inhibitory activity against both IMP-1 and VIM-2. The inhibition constants (*K*_i_) of DTPA-ADCA against IMP-1 and VIM-2 were estimated to be 30 and 0.35 μM, respectively. The *K*_i_ values of TNBM have been reported to be 30 μM for IMP-1 and 0.019 μM for VIM-2 (22). These results indicate that, at the enzymatic level, DTPA-ADCA exhibits inhibitory activity against IMP-1 comparable to that of TNBM.

In combination with MEPM against KP1, DTPA-ADCA showed a synergistic effect, whereas TNBM did not improve MEPM susceptibility. One possible explanation is that TNBM was ineffective against the KP1 strain used in this study because of limited membrane permeability or other factors affecting intracellular access. In contrast, DTPA-ADCA exhibited weaker inhibitory activity against VIM-2 than TNBM, as reflected by its higher *K*_i_ value. However, although evaluated only by IC_50_ assays, DTPA-AMOX showed the strongest inhibitory activity against VIM-2 among the synthesized DTPA-β-lactams. Further detailed analyses, including studies using VIM-2-producing strains, will therefore be necessary to clarify the potential of DTPA-AMOX.

Experiments examining the effect of zinc on IMP-1 inhibition by DTPA-ADCA suggested that DTPA-ADCA strongly interacts with the zinc-binding region of IMP-1. In this respect, the inhibitory mechanism of DTPA-ADCA appears to resemble that of TNBM, which also targets the active-site zinc ions of MBLs. In contrast, analysis of VIM-2 after treatment with DTPA-ADCA demonstrated that zinc ions were removed from the enzyme. This inhibitory mode is similar to that of chelating compounds such as DTPA and EDTA. This observation is consistent with previous reports describing differences in the sensitivity of IMP-1 and VIM-2 to chelating agents (23–25). For instance, Laraki et al. reported that metal chelators inhibit IMP-1 via a pseudo-first-order reaction, forming a ternary complex through binding to the active-site zinc ions (23). In contrast, EDTA, a representative metal chelator, has been shown to inhibit VIM-2 by removing its active-site zinc ions (25). On the other hand, IC_50_ assays demonstrated that the DTPA-β-lactam inhibited VIM-2 at lower concentrations than the simple chelating compounds DTPA and EDTA. These findings suggest that DTPA-β-lactams may access the active site more efficiently and subsequently promote zinc removal more effectively than simple chelators.

In addition to DTPA-β-lactams, we also found that DTPA-tryptophan enhanced the susceptibility of KP1 against MEPM, which indicated the inhibition of IMP-1 in bacteria (Fig. S7). Previous studies have reported that indole-2-carboxylates function as Type II MBL inhibitors by mimicking the proposed initial binding mode of intact β-lactam structure to the enzyme active sites (26). Although our synthesized DTPA-tryptophan contains an indole moiety, it lacks a C-2 carboxylate group. Nevertheless, DTPA-tryptophan contains multiple carboxylate groups in the vicinity of the indole ring, which may contribute to enhanced binding to MBL in a manner analogous to C-2 carboxylates. Further investigation is required to determine whether DTPA-tryptophan inhibits MBL via a mechanism similar to that of indole-2-carboxylates.

The synthesized DTPA-β-lactams were effective against a broad range of gram-negative bacteria expressing IMP-1, including *E. coli*, *K. pneumoniae*, *C. amalonaticus*, and *C. freundii*, significantly enhancing their susceptibility to carbapenems. This finding indicates that DTPA-β-lactams are taken up by gram-negative bacteria and exert their inhibitory effects in the periplasmic space. In contrast, TNBM did not enhance susceptibility in KP1, one of the tested strains. This is consistent with previous reports indicating that TNBM exhibits weak inhibitory activity against IMP-1 (27, 28). Moreover, recent studies have shown that mutations in NDM and VIM enzymes can confer resistance to inhibition by TNBM (29–31). These findings underscore the importance of developing a diverse repertoire of MBL inhibitors with varying mechanisms of action.

Notably, DTPA-ADCA demonstrated a potent therapeutic effect against KP1 *in vivo*. A single administration of DTPA-ADCA significantly enhanced the pharmacological efficacy of MEPM in a lethal *K. pneumoniae* infection model. Furthermore, the bacterial burden in infected animals was markedly reduced by the combination of DTPA-ADCA and MEPM, indicating a direct bactericidal effect.

Metal-chelating compounds have been considered promising candidates for MBL inhibitor development (32). Aspergillomarasmine A (AMA), an amino acid–derived fungal secondary metabolite, has been shown to reverse carbapenem resistance in animal infection models (33, 34). In that study, a single dose of AMA combined with MEPM significantly improved survival and reduced bacterial loads in the spleen and liver in a lethal peritoneal infection model with *K. pneumoniae*. Similarly, our findings demonstrate that the combination of DTPA-ADCA and MEPM is effective in an *in vivo* infection model. Chelator-β-lactam conjugates offer structural flexibility, allowing various combinations of chelators and β-lactam antibiotics. This modularity enables the rational design of chelator-β-lactams tailored for enhanced efficacy against specific bacterial targets.

Chelating compounds are promising candidates as MBL inhibitors; however, their potential toxicity due to off-target effects remains a concern (35, 36). In particular, since humans possess numerous metalloenzymes (37), it is essential to develop compounds that selectively inhibit MBL without affecting host metalloenzymes. Our present findings demonstrate that DTPA-β-lactams inhibit MBL at lower concentrations compared to DTPA or EDTA, and further enhance the efficacy of MEPM *in vivo*. Moreover, our previous studies have shown that DTPA-AMOX does not exhibit significant cytotoxicity in cultured human cells, even at concentrations up to 400 μM (19). These results suggest that further optimization of chelator-β-lactam conjugates to enhance MBL selectivity may contribute to the development of safer and more effective therapeutic agents.

In conclusion, DTPA-β-lactams were shown to restore carbapenem susceptibility in multidrug-resistant Gram-negative bacteria expressing MBL. These gram-negative bacteria include carbapenem-resistant Enterobacteriaceae (CRE), such as *K. pneumoniae*, *E. coli*, and *C. freundii*, which are clinically important pathogens in human infections. According to reports from the Centers for Disease Control and Prevention (CDC) and the World Health Organization (WHO), carbapenemase-producing Enterobacteriaceae (CPE) represent one of the most critical antimicrobial resistance threats (38). CPE has emerged as a major global public health concern due to the difficulty in treating infections caused by these organisms in healthcare settings (39). The combination of DTPA-β-lactams with carbapenems holds promise as a future therapeutic strategy against CRE.

## Data Availability

All data generated or analyzed during this study are included in this article. For further inquiries, please contact the corresponding author.

## SUPPLEMENTAL MATERIAL

Supplemental Material spectrum.02532-25-s0001.pdf Table S1; Fig. S1 to S8.

ASM does not own the copyrights to Supplemental Material that may be linked to, or accessed through, an article. The authors have granted ASM a non-exclusive, world-wide license to publish the Supplemental Material files. Please contact the corresponding author directly for reuse.
