# Supplementary material for: Chelate-conjugated β-lactams resensitize metallo-β-lactamase IMP-1-producing gram-negative bacteria against carbapenem antibiotics
Source: Microbiol Spectr. 2026 Jul 23;14(9):e02532-25. doi: 10.1128/spectrum.02532-25 (PMC13532291; doi:10.1128/spectrum.02532-25)
Supplement: Supplementary file 1 [file spectrum.02532-25-s0001.pdf]

Table S1. Bacterial strains and plasmids used in this study

Bacterial strains	Source
IMP-1-expressing *K. pneumoniae*-1 (KP1)	Kumamoto University Hospital
IMP-1-expressing *K. pneumoniae*-2 (KP2)	Kumamoto University Hospital
IMP-1-expressing *E. coli*	Kumamoto University Hospital
IMP-1-expressing *C. amalonaticus*	Kumamoto University Hospital
IMP-1-expressing *C. freundii*	Kumamoto University Hospital
VIM-2-expressing *P. aeruginosa*	Kumamoto University Hospital
E. coli DH5α	Laboratory stock
pQE-IMP-1-expressing *E. coli* DH5α	This study
pQE-VIM-2-expressing *E. coli* DH5α	This study
E. coli BL21(DE3) pLysS	Laboratory stock
pQE-IMP-1-expressing *E. coli* BL21(DE3) pLysS	This study
pQE-VIM-2-expressing *E. coli* BL21(DE3) pLysS	This study
Plasmids	Source
pQE80L	Qiagen
pQE80L-IMP-1	This study
pQE80L-VIM-2	This study

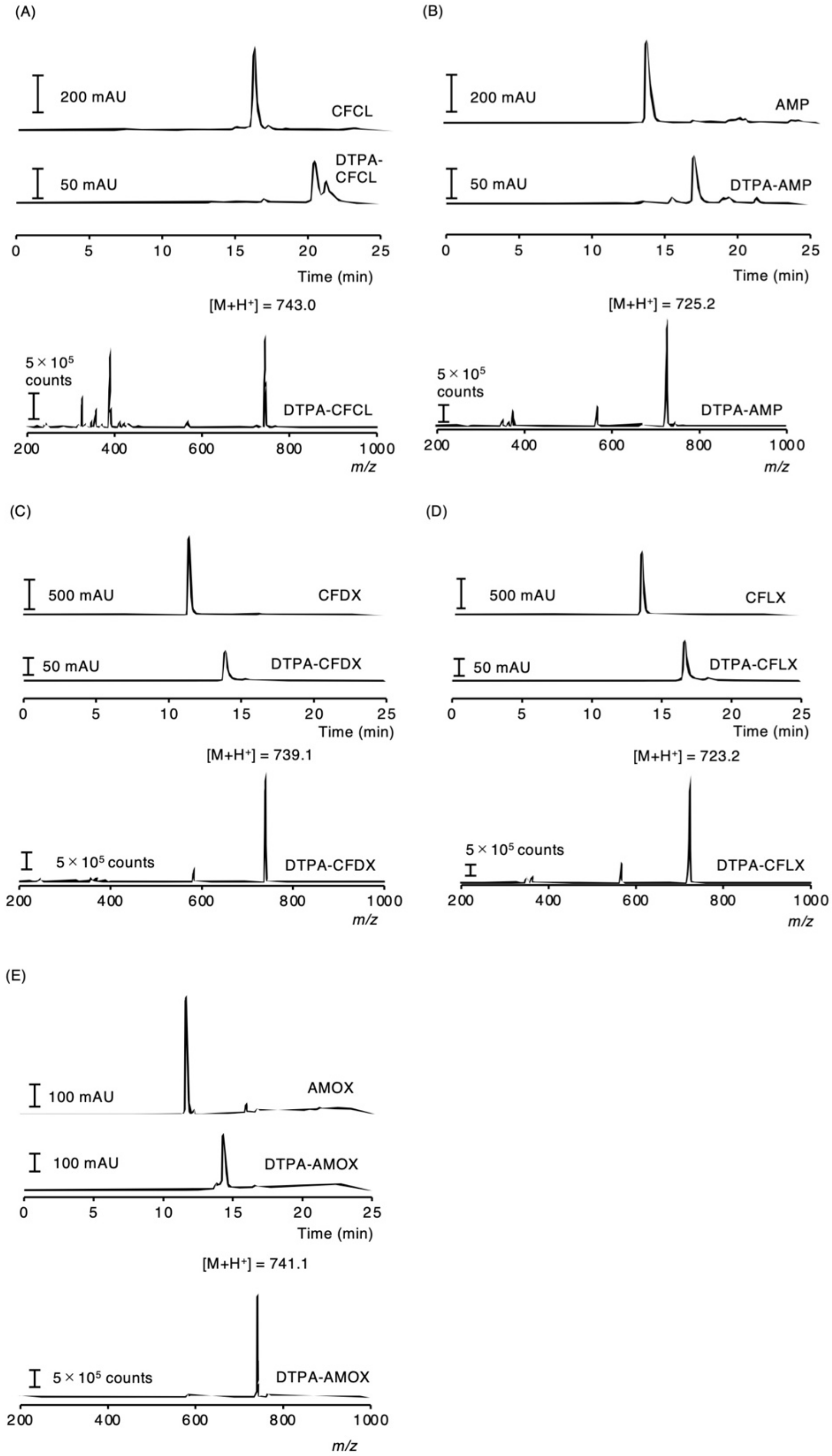

Fig. S1. Characterization of DTPA–β-lactams. HPLC chromatograms of the parent β-lactams (upper panels) and their DTPA-conjugated forms (lower panels). (A) CFCL, (B) AMP, (C) CFDX, (D) CFLX, and (E) AMOX. Mass spectra of the DTPA–β-lactams are shown below the HPLC chromatograms.

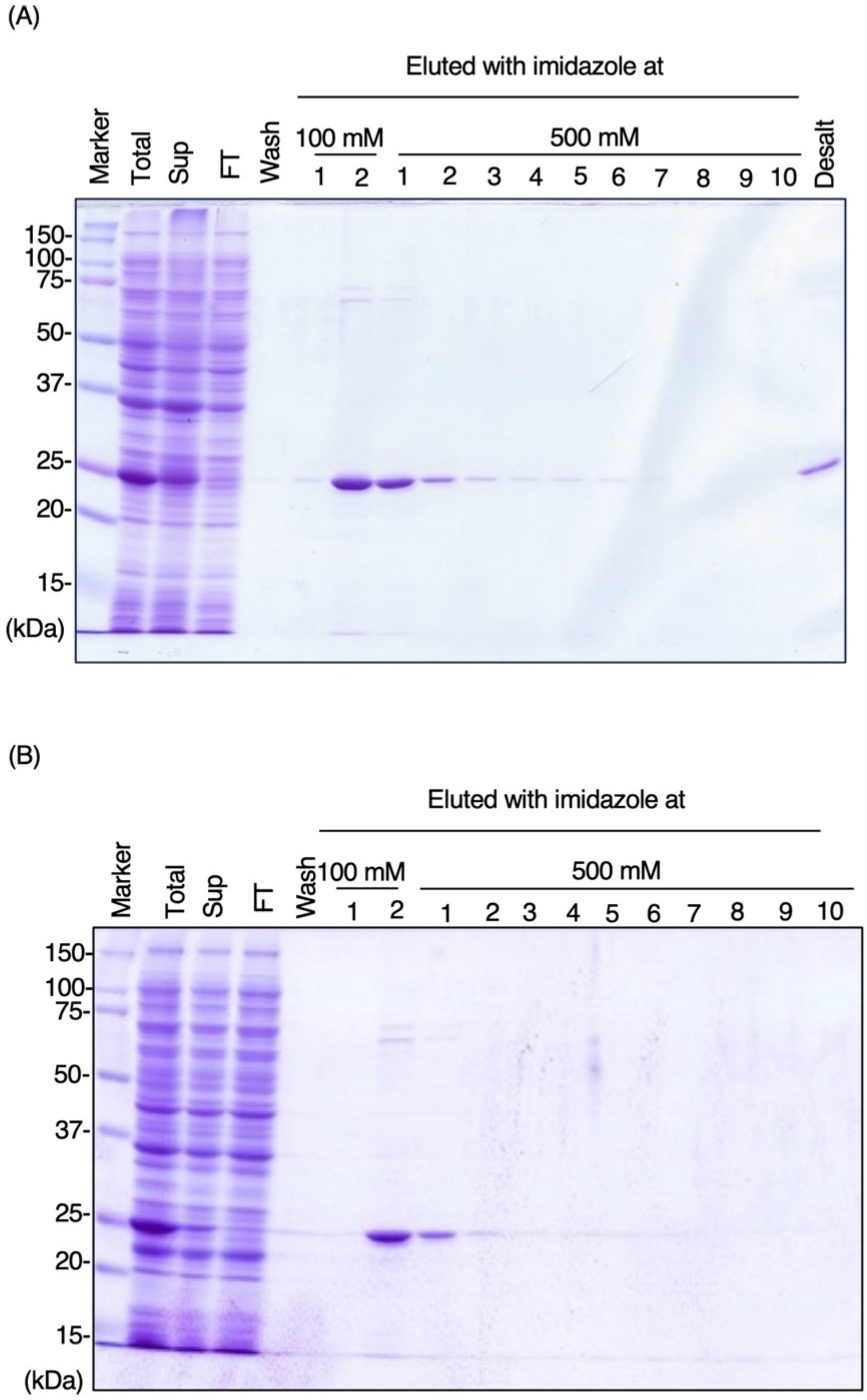

Fig. S2. Purification of recombinant His-tagged IMP-1 and VIM-2 using Ni-NTA agarose affinity chromatography. The images show the His-tagged IMP-1 (upper panels) and His-tagged VIM-2 (lower panels) at each stage of purification. Sup, supernatant; FT, flow-through; numbers indicate elution fractions.

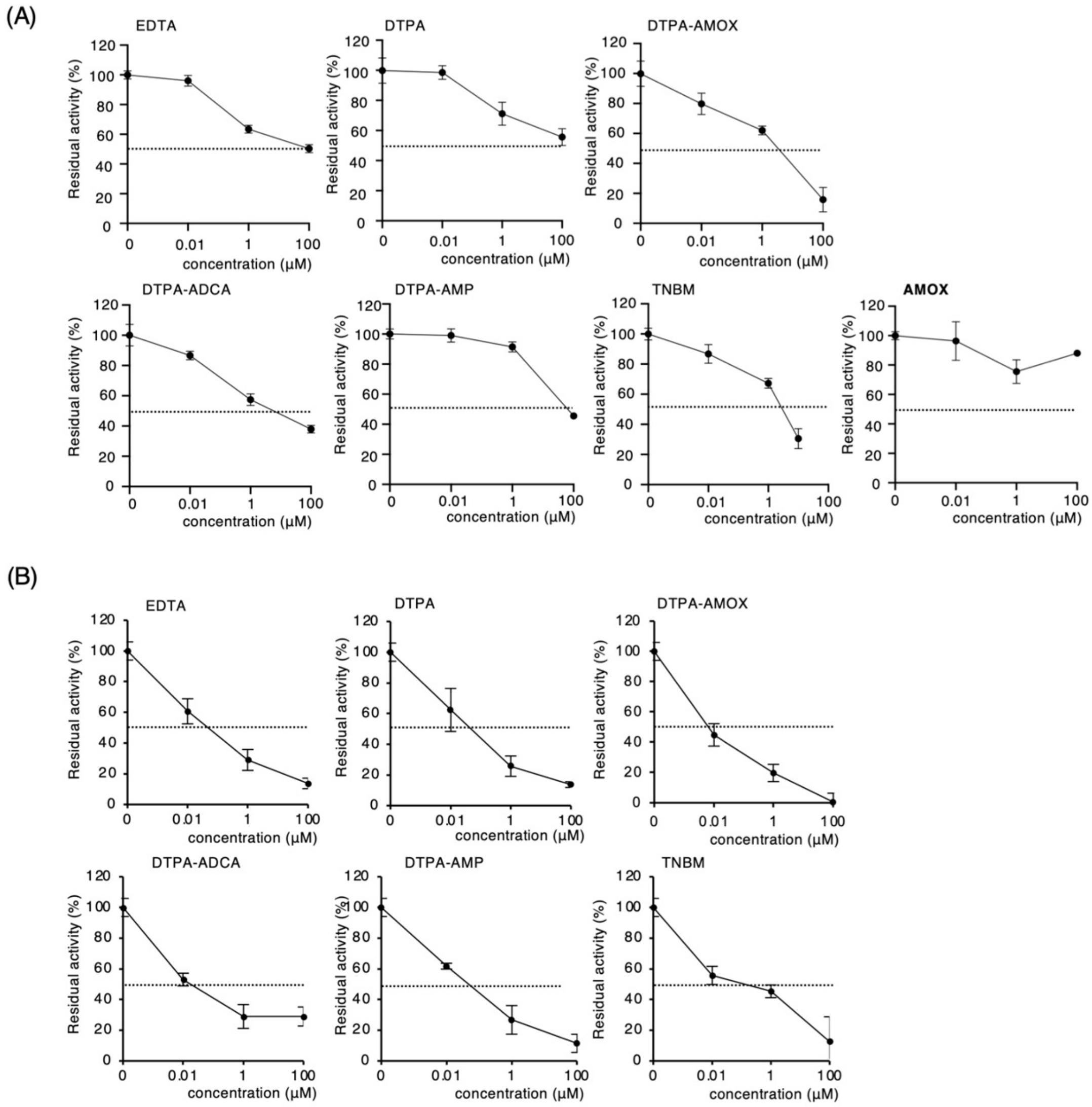

Fig. S3. Effects of DTPA–β-lactams on the enzymatic activities of IMP-1 (A) and VIM-2 (B). IMP-1 (100 nM) or VIM-2 (100 nM) was preincubated with the indicated concentrations of the compounds at 30 °C for 5 min in 50 mM HEPES buffer (pH 7.5) containing 200 mM NaCl, followed by the addition of imipenem (200 μM). Enzymatic activities of IMP-1 and VIM-2 were determined by monitoring imipenem hydrolysis as a decrease in absorbance at 298 nm. Data are presented as the mean ± SD (n = 3). The IC_{50} values were estimated from the plots as the concentrations required to inhibit enzyme activity by 50%.

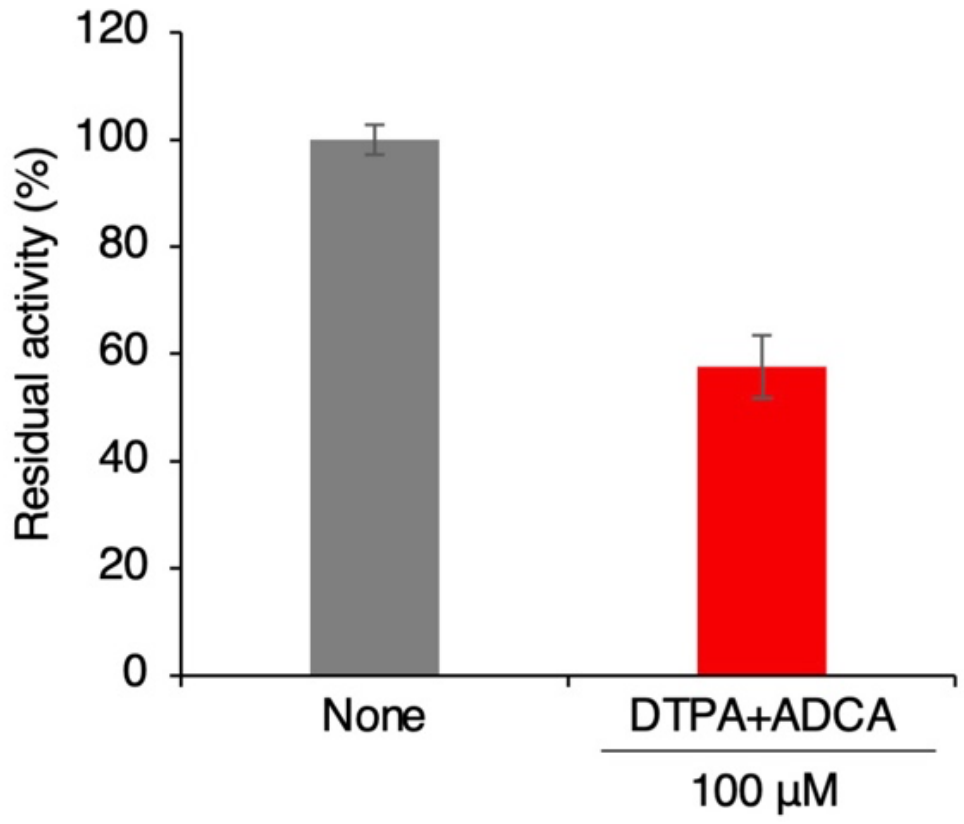

Fig. S4. Effect of the combination of DTPA and ADCA on the enzymatic activity of IMP-1. As described in Figure S3, His-tagged IMP-1 was preincubated with a combination of ADCA (100 μM) and DTPA (100 μM), followed by the addition of imipenem. Residual enzymatic activity was evaluated by monitoring absorbance at 298 nm. Data are presented as the mean ± SD (n = 3).

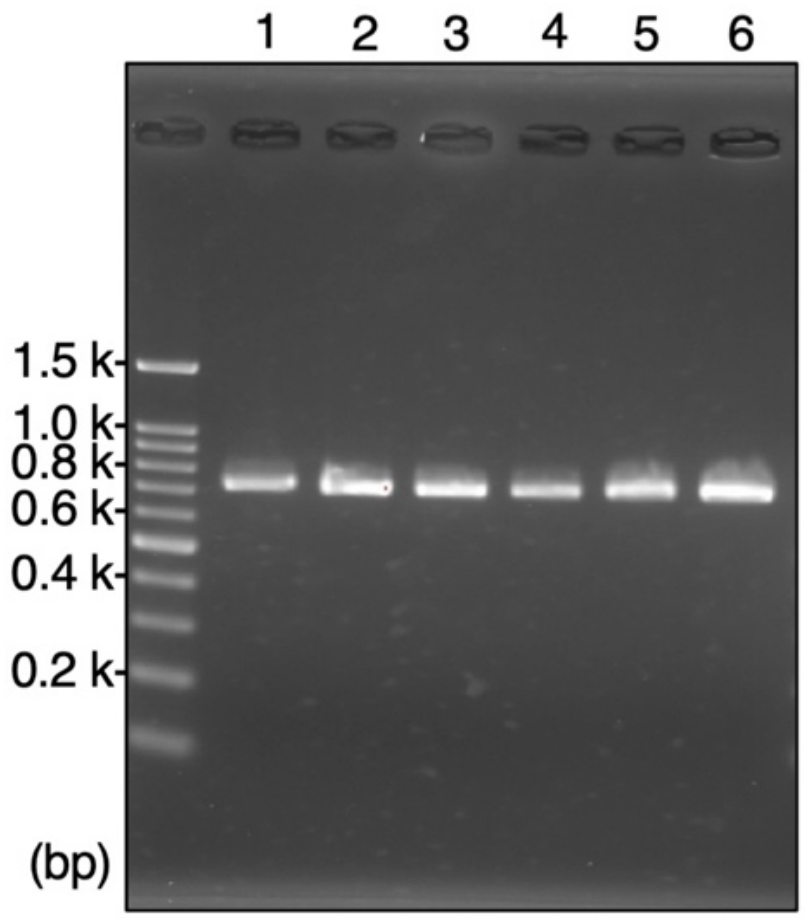

Fig. S5. Determination of *bla$_{IMP-1}$*. Lane 1, positive control; Lane 2, KP1; Lane 3, KP2; Lane 4, *E. coli*; Lane 5, *C. freundii*; Lane 6, *C. alonaticus*.

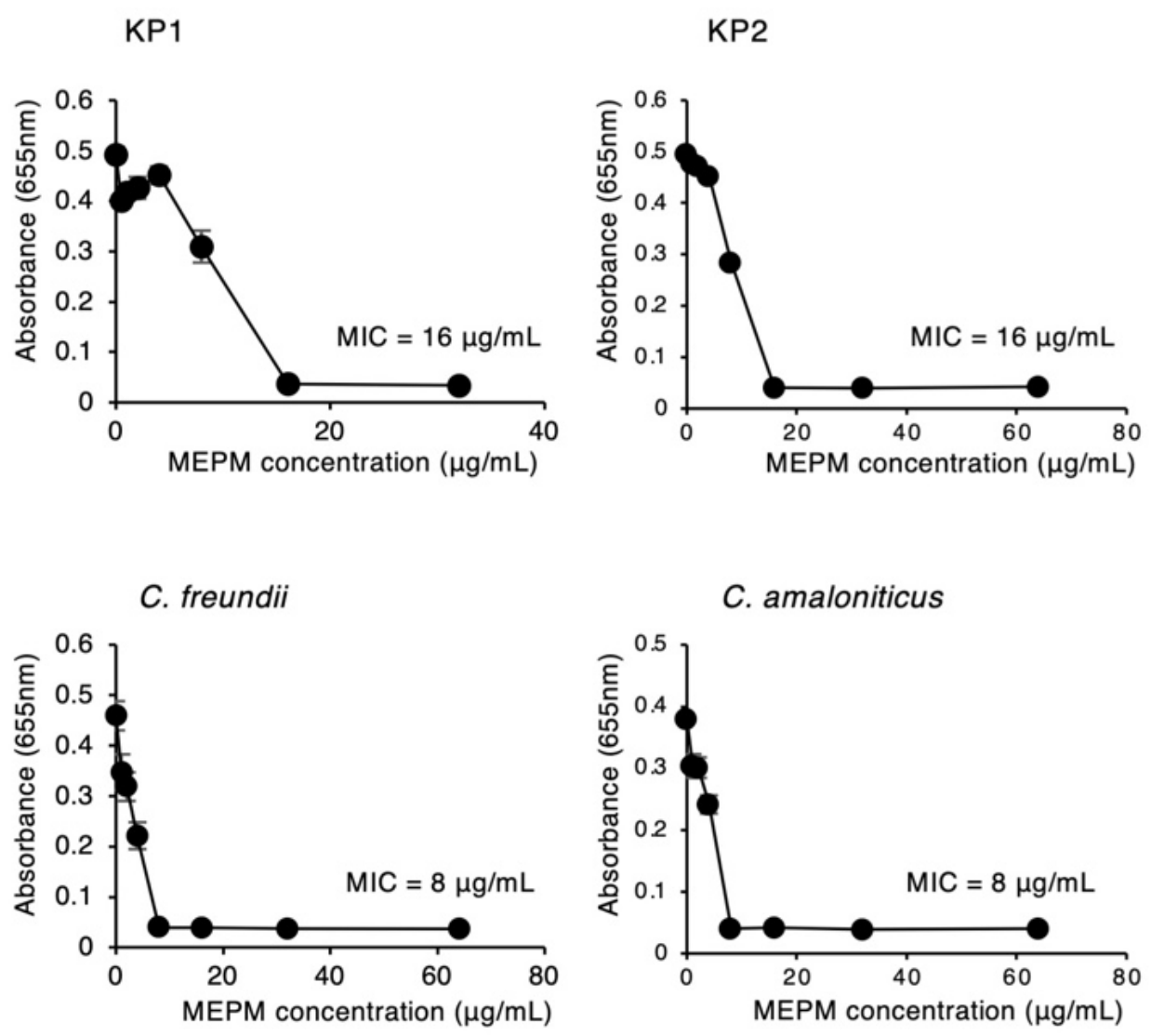

Fig. S6. Determination of the MIC of meropenem against IMP-1-expressing clinical isolates. Clinical isolates were incubated with indicated concentrations of meropenem (MEPM) for 20 hours at 37°C. Bacterial growth was determined by measuring absorbance at 655 nm. Data are means ± SD (n=3).

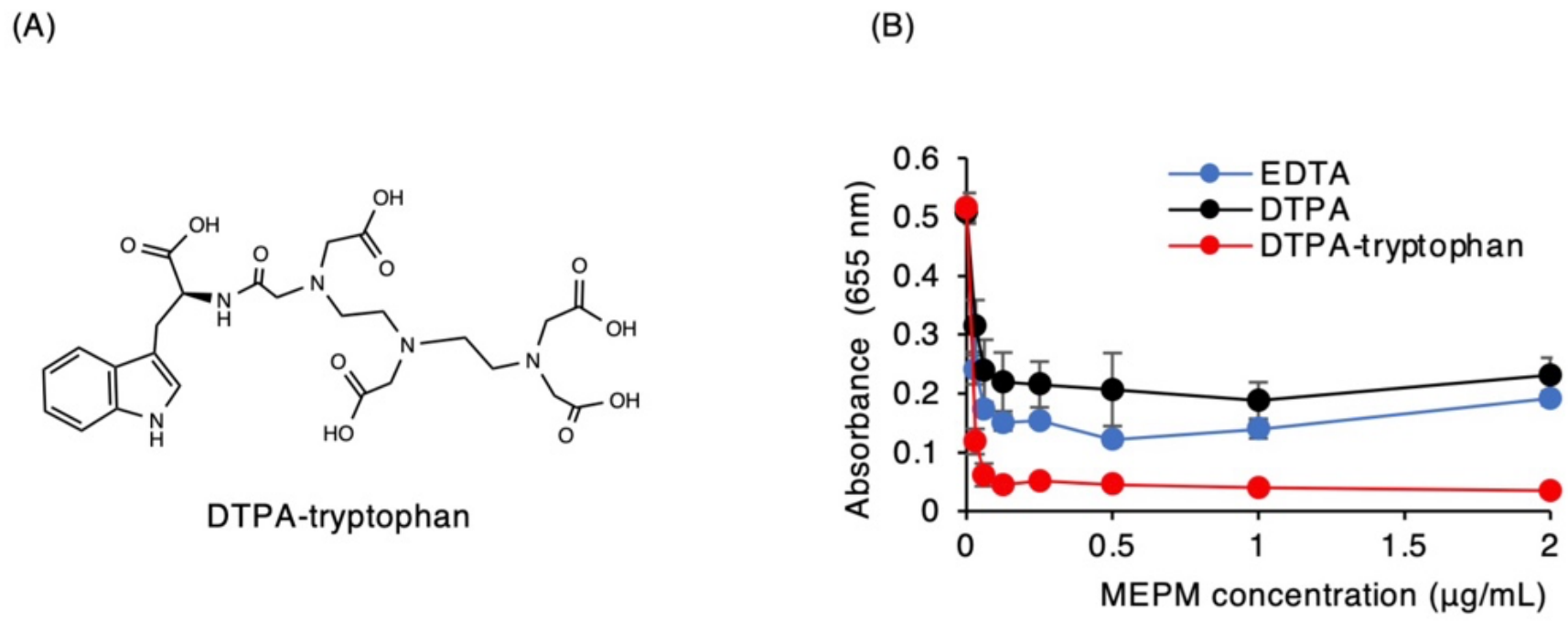

Fig. S7. Antibacterial effect of DTPA-tryptophan on the meropenem sensitivity of KP1. (A) Chemical structure of DTPA-tryptophan. (B) KP1 were cultured in LB medium containing 25 μM EDTA, DTPA, or DTPA-tryptophan, along with varying concentrations of meropenem (MEPM) as indicated. After 20 hours, bacterial growth was determined by measuring turbidity at 655 nm. Data are means ± SD. Data are means ± SD (n=3).

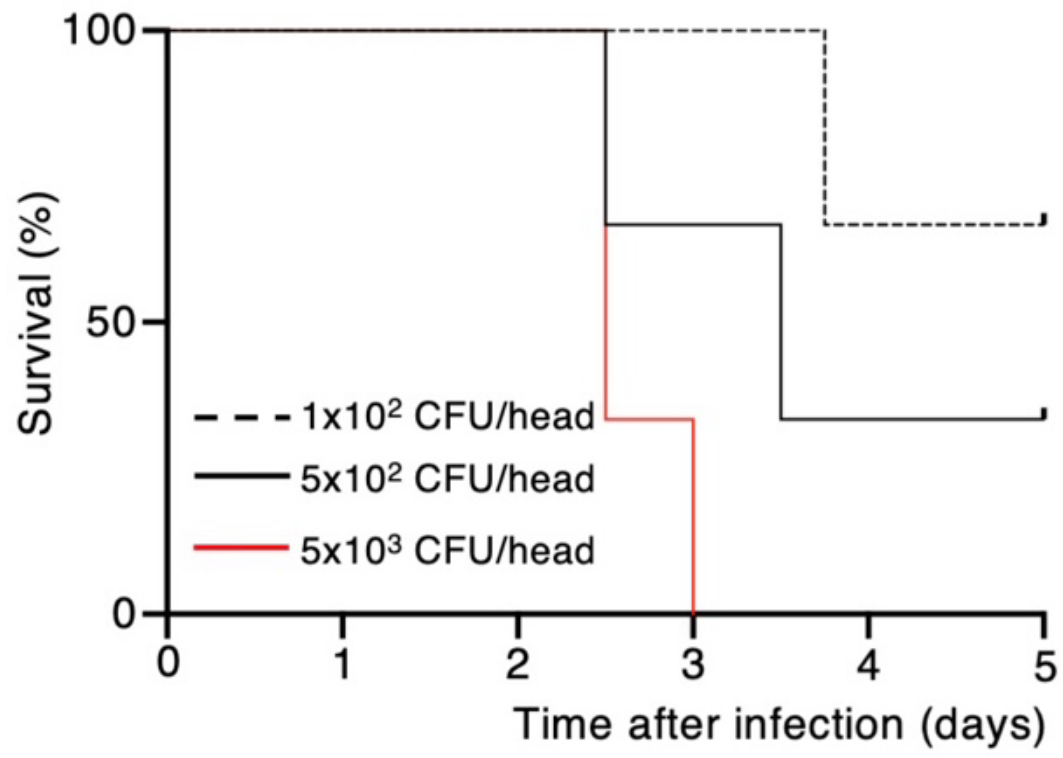

Fig. S8. Survival curves of mice following infection with *Klebsiella pneumoniae* at different inoculation doses. Four-week-old ddY mice were intraperitoneally inoculated with the indicated doses of KP1. Survival curves were generated using the Kaplan–Meier method ($n = 3$).
